# Portacaval anastomosis promotes fragmentation of mitochondrial network in the cerebellum of male rats

**DOI:** 10.1007/s11011-025-01705-8

**Published:** 2025-09-24

**Authors:** Mayra López-Cervantes, Andrés Quintanar-Stephano, Rogelio Hérnandez-Pando, Raúl Aguilar-Roblero, Jorge Larriva-Sahd, Olivia Vázquez-Martínez, Gema Martínez-Cabrera, Mauricio Díaz-Muñoz

**Affiliations:** 1 Cellular and Molecular Neurobiology Department, Neurobiology Institute, campus UNAM-Juriquilla, Querétaro, 76230 QRO México; 2 Physiology and Pharmacology Department, Basic Science Center, UAA, Aguascalientes, AGS 20100 México; 3 Sección de Patología Experimental, Departamento de Patología, Instituto Nacional de Ciencias Médicas y Nutrición Salvador Zubirán, México City, México; 4 Neuroscience Division, Cellular Physiology Institute, México City, 04510 México; 5 Neurophysiology and Neurodevelopment Department, Neurobiology Institute, campus UNAM-Juriquilla, Querétaro, QRO 76230 México

**Keywords:** Spongiform neurodegeneration, Hepatic encephalopathy, Oxidative stress, Ultrastructure

## Abstract

Portacaval anastomosis (PCA) is a model for hypometabolic liver dysfunction. Spongiform neurodegeneration has been detected in the cerebellum of PCA rats 13 weeks after surgery. This report characterizes the damage associated with spongiform degeneration by studying mitochondrial, ultrastructural, and oxidative changes in the molecular, Purkinje, and granular layers of the cerebellar cortex. Morphometry by electron microscopy determined an increase in mitochondrial presence in PCA rats. In parallel, mitochondria displayed smaller size, diminished interconnectivity, and decreased elongation. Fluorescent probes revealed that PCA cerebellar mitochondria showed a reduction in membrane potential (ΔΨ) alongside a rise in superoxide levels. In contrast, the calcium content exhibited variability across the three cerebellar layers. In addition, an elevation of intracellular reactive oxygen species in the cerebellar cortex was detected. The measurement of TBARS, conjugated dienes, and total antioxidant activity confirmed the presence of oxidative stress in the PCA cerebella. The increased number of smaller mitochondria was accompanied by an altered equilibrium between mitochondrial fission and fusion markers in PCA rats: increased FIS1 and p-DRP1, as well as OPA1, but decreased MFN1. Immunohistochemical analyses of these markers indicated that the molecular layer was the most affected in the cerebellum of PCA rats. In conclusion, we characterized the active cerebellar damage associated with dysregulated mitochondrial activity accompanied by an evident pro-oxidative condition. Ultrastructural analysis helped to strengthen the depiction of the mitochondrial and biochemical alterations associated with the spongiform vacuolization observed in the PCA cerebellar cortex, especially within the molecular layer.

**Graphical abstract:**

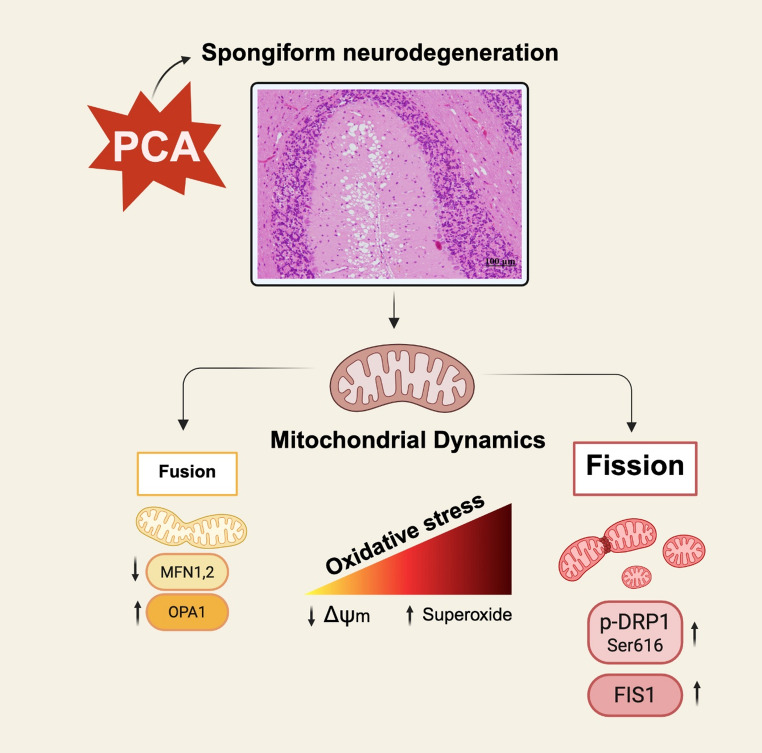

**Supplementary Information:**

The online version contains supplementary material available at 10.1007/s11011-025-01705-8.

## Introduction

According to pragmatic and conceptual considerations made by the International Society for Hepatic Encephalopathy and Nitrogen Metabolism Commission (ISHEN), end-to-side portacaval anastomosis (PCA) is a suitable animal model to study clinical episodes of hepatic encephalopathy (HE) (Butterworth et al. 2009; DeMorrow et al. 2021). PCA in experimental animals serves as a useful research model for exploring physiopathological mechanisms underlying cellular alterations associated with encephalopathy events that occur alongside hyperammonemia during liver hypofunctional states (Butterworth 1993; Claeys et al. 2021; Su et al. 2015). In contrast, the use of a surgical connection between portal and inferior vena cava veins to control variceal bleeding in liver failure patients has become less common due to the emergence of more effective clinical treatments, such as the transjugular intrahepatic portosystemic shunt (Neong et al. 2019). Our group recently rediscovered a striking feature in PCA rats, initially reported by Cavanagh and colleagues in 1972: cerebellar spongiform neurodegeneration occurring after 16 weeks post-surgical procedure (Cavanagh et al. 1972). In that article, the authors conducted a longitudinal characterization of the vacuolizing process exclusively in the molecular layer of the cerebellum. The authors suggested that spongiform formation, as indicated by histopathological analysis with electron microscopy images, represented a focal swelling of glial processes close to the dendrites of Purkinje neurons and stellate cells, without the presence of phagocytic entities. They also observed that regions next to the vacuoles were enriched with Alzheimer type II astrocytes and in close apposition to zones lacking Purkinje neurons (Cavanagh et al. 1972).

We enriched these initial findings and extended the histopathological characterization with in vivo magnetic resonance spectroscopy, behavioral studies, and other markers of cellular damage (López-Cervantes et al. 2021). Our key findings in 13-week-old PCA male rats were: a conspicuous vacuolar/spongiform deterioration detected only in the molecular layer of the cerebellum; significant loss of Purkinje neurons; disorganized structure in Glial fibrillary acidic protein GFAP-positive cells in the granular and molecular layers; decreased number of nuclei within the granular layer; activation of microglia; enhancement in glutamine synthetase presence, and cellular markers of edema and inflammation; changes in levels of glutamate, glutamine, N-acetylaspartate, taurine, creatine phosphate; deficits in the rotarod test and in locomotor activity performance. Given that different cellular alterations were observed in the granular and molecular layers, as well as in Purkinje neurons, the aim of the present report was to further characterize the regional cerebellar cortex damage associated with PCA.

One of the most important organelles for preserving cellular physiology and integrity is the mitochondrion (Picca et al. 2021). Mitochondria exhibit plastic membrane adaptations in response to physiological and pathological stimuli, and are highly dynamic entities with the reversible capability of forming complex tubular systems, and their network is regulated by fission and fusion events involving the inner and outer mitochondrial membranes (IMM and OMM, respectively) (Bennett et al. 2022). DRP1, a dynamin GTPase complex, orchestrates mitochondrial morphology. Key factors for mitochondrial fusion processes include mitofusins (MFN) 1 and 2 and optic atrophy 1 (OPA1) protein (Giacomello et al. 2020; Grel et al. 2023). Mitochondrial fission can occur in the midzone, signaling growth and healthy mitochondria, and in the peripheral zone, indicating damage or partially degenerated mitochondria (Grel et al. 2023). When one end of the mitochondrion shows a decrease in membrane potential with an increase in reactive oxygen species (ROS), cytosolic dynamin- related protein 1 (DRP1) is assembled at the site of division, forming a ring that mechanically separates the organelle. Numerous OMM proteins help recruit DRP1, such as FIS1, MFF, and MiD 49 and 50. FIS1 is an important receptor for DRP1. Furthermore, phosphorylation plays a fundamental role in the dynamics of DRP1. Phosphorylation at serine 616 drives mitochondrial fragmentation, whereas phosphorylation at serine 637 inhibits DRP1 and prevents mitochondrial separation. Overall, mitochondrial dynamics is essential for maintaining effective mitochondrial quality control and adequate cellular responses to stress (Youle and Van Der Bliek 2012).

In the nervous system, the proper functioning of the unit formed by neurons (mainly oxidative) and astrocytes (primarily glycolytic) requires the coordination of their mitochondrial metabolic activities (Riske et al. 2017).

The mitochondrial system plays a significant role in influencing the pro-oxidant status of the cell. The equilibrium of pro-oxidant reactions and antioxidant defenses is finely regulated by the NAD^+^/NADH ratio and the biochemical electron fluxes in the cytoplasmic and mitochondrial compartments. This equilibrium is vital for the correct synchronization of biochemical oxidations that govern the redox state, a key regulator of metabolic networks (Hu et al. 2020). Indeed, the disruption of this equilibrium can promote pathological conditions that compromise cellular integrity, such as oxidative stress and inflammation (Angelova et al. 2018; Cauli et al. 2007). In this context, Kosenko et al. reported that in PCA rats 4 weeks post-surgery, cerebellar mitochondria exhibited higher superoxide production than other brain regions. This pro-oxidant action was correlated with ammonia levels (Kosenko et al. 2017).

Following 13 weeks of PCA in male rats, we employed ultrastructural morphometric parameters to assess mitochondrial presence and utilized fluorescent probes to characterize mitochondrial membrane potential, superoxide generation, calcium content, and intracellular ROS production in the molecular and granular layers, as well as in Purkinje neurons of the cerebellar cortex. The pro-oxidant state of the cerebellum was further assessed with biochemical techniques such as conjugated dienes, thiobarbituric reactive substance assay (TBARS), glutathione peroxidase activity, and antioxidant capacity. In addition, we evaluated the presence of markers associated with mitochondrial fusion and fission events using western blot and immunohistochemistry.

## Materials and methods

### Animal protocol

The research protocol that involves control and experimental groups has been previously described (López-Cervantes et al. 2021; Vázquez-Martínez et al. 2019). Briefly, young adult male Wistar rats (∼280 g and ∼2 months old) were randomly subjected to PCA or a sham surgery (Sham group). The animals were housed in individual cages at room temperature and with ad libitum access to food until full recovery. All procedures adhered to the institutional guidelines for the care and use of animals in biomedical experimentation, as well as the international ethical standards established by the National Autonomous University of Mexico (UNAM). Approval was granted by the Research Ethics Committee of the Neurobiology Institute, UNAM (protocol 081.A). The Official Mexican Regulation NOM-062-ZOO-1999/SAGARPA was also considered.

### Surgery

The procedure by Lee et al. (Lee et al. 1974) for PCA was followed with certain modifications as previously reported (López-Cervantes et al. 2021; Vázquez-Martínez et al. 2019). Briefly, around 30 rats were put under anesthesia with Ketamine (ANESKET^®^, 1000 mg 10 ml-1, PiSA Agropecuaria, MEX)/xylazine (PROCIN^®^, 20 mg ml-1, PiSA Agropecuaria, MEX), and a laparotomy was performed to access the abdominal organs. The portal vein was dissected and occluded. The extreme of the portal vein was then connected to a window on the inferior portal vein that was previously obstructed with surgical clips. To optimize the survival of the PCA, the microsurgery was completed in less than 20 min to avoid compromising the survival of the animals. 24 sham-operated rats were subjected to the same procedure (until the use of the surgical clips) but without cutting any blood vessel. The animals (2–3 rats) were kept in a recovery box with controlled temperature. To prevent infections, rats received an intramuscular (IM) injection of piperacillin (6,000 IU). For analgesia, sodium metamizole (10 mg/kg IM) was administered once daily for three days. Surviving animals 24 PCA rats (~ 85% of the total operated animals) participated in the experiments.

### Tissue sampling

After a 13-week postoperative period, rats were euthanized with an intraperitoneal injection of ketamine (70 mg/kg) and xylazine (8 mg/kg). Four types of experiments were conducted: (1) Tissue sampling for electron microscopy(3 sham and 3 PCA rats); (2) Ex-vivo tests using cerebellar slices for incubation with fluorescent probes (for all experiments around 10 sham and 10 PCA rats); (3) Immunohistochemical assays involving trans-cardiac perfusion to fix the organs from unresponsive animals (5 sham and 5 PCA rats); (4) Homogenate cerebellum sample for biochemical assays and western blot (~ 6 sham and ~ 6 PCA rats) (Gage et al. 2012). For fluorescent and immunohistochemical experiments, the cerebella were treated as previously reported (López-Cervantes et al. 2021). A cryostat (Leica CM3050 S) was used to obtain 7–8 consecutive sagittal sections of the cerebellum (from the vermis) at ∼30 μm thickness and a temperature of −30 °C.

### Transmission electron microscopy

Cerebellar samples were processed as previously reported by Larriva-Sahd, 2006 and Paredes and Larriva-Sahd, 2010. Briefly, eight control and eight PCA rats were deeply anesthetized and intracardially perfused with 200 ml of Karnovsky’s fixative (4% paraformaldehyde, 2.5% glutaraldehyde in 0.10 M cacodylate buffer, pH 7.3–7.4. Each brain was then dissected. Then, tissue samples were post-fixed for 1 h in 1% osmium tetroxide, dehydrated, and flat-embedded in epoxy resins. Semi-thin Sects. (300–400 nm) were obtained using a Leica Ultramicrotome. Ultrathin sections measuring 70–90 nm were mounted on 200-mesh copper grids. The sections were treated with aqueous solutions of uranium acetate and lead citrate and then observed under a JEOL 1010 electron microscope.

Determination of mitochondrial parameters. Electron microscopy images were used to analyze subcellular entities identified as mitochondria, characterized by a double membrane system and cristae. The number of mitochondria, along with their area and perimeter, was quantified with image analysis software (NIH, USA). We analyzed 150–250 mitochondria in the molecular, granular, and Purkinje cell layers from 10 micrographs per group.

Mitochondria morphology was characterized according to Wiemerslage and Lee (2016). The mean area and number of mitochondria were used as indicators of mitochondrial presence. The following four parameters were considered for mitochondrial morphology quantification: mitochondrial quantity, size, interconnectivity, and elongation. The circularity was calculated using the pixel area and the estimated perimeter. The interconnectivity score, which evaluates the physical connections and fragmented status of the mitochondrial network, as well as the elongation score, defined as the inverse value of circularity, were calculated as reported by Wiemerslage and Lee, 2016 with the following formulas:

$$\:interconnectivity=\frac{mean\:area}{mean\:perimeter}$$

$$\:elongation=\:\frac{1}{circularity}$$

$$\:circularity=4*\pi\:*\left(\frac{mean\:area}{{mean\:perimeter}^{2}}\right)$$

Interconnectivity describes the connections between mitochondria, being an indicator of mitochondrial networking. High score of interconnectivity indicate that mitochondria have more physical connections, while lower scores signify that mitochondria are more fragmented. Elongation refers to the shape of organelle, considering the value of 1 as a circle, associated with mitochondrial fission. Values above 1 correlate to increased fusion.

### Fluorescent probes

The following fluorescent probes were used in accordance with previously reported protocols:1) MitoTracker Red (100 nM) to measure mitochondrial membrane potential (ΔѰ); 2) Rhod-2 (4 µM) following treatment with NaBH_4_ to enhance the mitochondrial calcium signal (Dihydrorhod-2 AM) (Lee et al. 1974); 3) MitoSox (10 µM) to quantify mitochondrial superoxide; 4) dichlorofluorescein diacetate (DCFH-DA; 10 µM) to evaluate intracellular ROS (Zhang and Gao 2015). The protocol involved the following steps: Cerebellar slices (∼1 mm) from sham and PCA rats were obtained using a razor and a sagittal matrix for reference and then pre-incubated for 15 min with Krebs solution (in mM: NaCl 155, KCl 4.5, NaHCO_3_ 1.9, CaCl_2_ 2.4, D-Glucose 10, MgCl_2_ 2, HEPES 5) at pH 7.4, with constant oxygenation (95% O_2_ and 5% CO_2_) at 37 °C under dim light. Each fluorescent probe was added at the final concentration specified and incubated for an additional 60 min.

After incubation, the slices were fixed in 4% paraformaldehyde for 24 h and sequentially rehydrated in sucrose solutions (from 10 to 30%). Tissue samples were cut to a thickness of 14 μm utilizing a cryostat (3050 S Leica model). Individual coverslips were washed three times with cold phosphate buffer saline (PBS). Nuclei were stained by adding propidium iodide (dilution 1:1000, Invitrogen); peak of excitation wavelength (λ ex) at 535 nm, peak of emission wavelength (λ em) at 615 nm or SYTOX Green (dilution 1:3000, Invitrogen) detection: λ ex: 504 nm, λ em: 523 nm. Fluorescence was detected with a Zeiss LSM-780 DUO confocal microscope. Fluorescent parameters were for MitoTracker Red: λ ex 510 nm, λ em: 580 nm, MitoSox: λ ex: 488 nm, λ em: 510 nm, Rhod-2: λ ex 552 nm, λ em: 581 nm and DCFH-DA λ ex:488 nm, λ em: 525 nm.

### Mitochondrial dynamics

Western blotting and immunofluorescence techniques were used to evaluate specific factors involved in mitochondrial dynamics in the cerebellum of PCA rats.

Western blotting: Cerebellar homogenate sample proteins (50 µg) were separated into 7.5% −15% SDS-polyacrylamide electrophoresis gels, which were transferred to nitrocellulose membranes. Non-specific sites were blocked with 5% of milk-free fatty acids in a TBS1x solution at room temperature for 1 h. The membrane was incubated overnight at 4 °C with the corresponding primary antibody: rabbit anti-OPA1 (Abcam Cat# ab157457, RRID: AB_2864313; 1:1000), rabbit anti-MNF1 (Cell Signaling Technology Cat# 14739, RRID: AB_2744531; 1:500), and rabbit anti-MNF2 (Cell Signaling Technology Cat# 11925, RRID: AB_2750893; 1:1000) for mitochondrial fusion, rabbit anti-FIS1 (Boster Biological Technology Cat# A01932-2, RRID: AB_3081504; 1:1000), rabbit anti-DRP1 (Huabio Cat# HA500487, RRID: AB_3071572; 1:1000) and rabbit anti phosphorylated DRP1Ser616 (Cell Signaling Technology Cat# 4494, RRID: AB_11178659; 1:1000) were utilized for mitochondrial fission, and rabbit anti-GADPH (Cell Signaling Technology Cat# 2118, RRID: AB_561053; 1:1000) as loading control. Then, the membranes were washed with TBST and incubated with the secondary antibody: goat anti-rabbit IgG coupled to alkaline phosphatase (Abcam Cat# ab97048, RRID: AB_10680574; 1:3000). The membranes were scanned and analyzed using Fiji-ImageJ software.

Tissue immunofluorescence: Sagittal sections from cerebellar tissue (14 μm thickness) were washed three times with PBS 1x, pH 7.4, for 10 min per wash. The sections were subsequently blocked with PBS 1x, 0.2% Triton 100x, and 10% normal goat serum, and then incubated overnight at 4 °C with primary antibodies: rabbit anti-OPA1 (Abcam Cat# ab157457, RRID: AB_2864313; 1:250), rabbit anti-MNF2 Cell Signaling Technology Cat# 11925, RRID: AB_2750893;1:250), rabbit anti-rabbit anti-MNF1 (Cell Signaling Technology Cat# 14739, RRID: AB_2744531;1:250), rabbit anti-FIS1 (Boster Biological Technology Cat# A01932-2, RRID: AB_3081504; 1:250), rabbit anti-DRP1 (Huabio Cat# HA500487, RRID: AB_3071572; 1:250), and rabbit phospho-DRP1Ser616 (Cell Signaling Technology Cat# 4494, RRID: AB_11178659;1:250). The samples were washed with PBS three times for 5 min and then revealed with anti-Alexa 488 (Thermo Fisher Scientific Cat# A32731, RRID: AB_2633280; 1:300). Nuclei were dyed with propidium iodide (1:1000 dilution) and + RNase A (1:1000 dilution) for 15 min. The assembly was carried out with the vectashield solution. Ten representative images were taken for analysis. All images were acquired with a Zeiss LSM 780 DUO confocal microscope.

### Image processing

To quantify the fluorescent signal of each assay, 3–6 representative images were taken for each layer of the cerebellar cortex, with a 63x oil immersion objective with a 2x (Granular layer) and 1.8x (Purkinje layer) optical zoom on a Zeiss LSM 780 confocal microscope. For 2D images from 14 μm thickness, z-stacking was performed according to the recommended microscope settings (0.37 μm optical sections). All images were acquired using the same intensity parameters and exposure time settings. All quantifications were performed using Image J Fiji for Image J software (NIH, USA).

### Lipoperoxidation and conjugated dienes tests

The whole cerebellum was homogenized in buffer (225 mM saccharose, 10 mM Tris/HCl, 0.2% BSA, 0.3 mM EGTA; pH 7.4) using 10 to 15 passes with a DOUNCE homogenizer. Lipid peroxidation (LP) was determined using TBARS, as previously described in Vázquez-Martínez et al. (2019), to quantify malondialdehyde (MDA) and other aldehydes. Briefly, 3 mg of protein was incubated under acidic conditions and placed in a boiling water bath with 0.8% thiobarbituric acid. The pink color was extracted with a pyridine: butanol solution (15:1 v/v) and read at 532 nm. Conjugate dienes were quantified from 500 mg of protein by separating the lipid fraction with the Folch reagent (chloroform-methanol 2:1 v/v). Subsequently, the samples were dried at room temperature and reconstituted in hexane. The extract was read using a UV spectrophotometer at 233 nm (Vázquez-Martínez et al. 2019).

### Cupric reducing antioxidant capacity assay

The cupric reducing antioxidant capacity (CUPRAC) assay measures the total antioxidant capacity of both hydrophilic and hydrophobic samples (Apak et al. 2008). Briefly, a cerebellar homogenate was prepared using a 7.5 mM copper (II)-neocuproine (CU(II)-Nc) reagent as a chromogenic oxidant and a 10 mM ammonium acetate solution (1:1 v/v) at pH 7. The mixture was incubated for 20 min at 50 °C, and the absorbance of the resulting CU(I)-chelate from the redox reaction was measured at 450 nm. Data were expressed as equivalents of TROLOX (Apak et al. 2008).

### Glutathione peroxidase activity

Glutathione peroxidase activity was measured using a previously reported coupled reaction (Díaz-Muñoz et al. 1985). The cerebellum was homogenized in a solution containing 225 mM sucrose,10 mM Tris/HCl, 2% BSA, and 0.3 mM EGTA (pH 7.4). The samples were mixed with 50 mM K_2_HPO_4_, 10 mM EDTA, 1 nM NADPH, 1 mM sodium azide, 100 units/ml glutathione reductase enzyme, and 200 mM reduced glutathione. The final solution was carefully shaken for a few seconds, and the reaction was started with H_2_O_2_ 0.042% (w/w) and read at 340 nm every minute for 5 min. The activity was calculated as micromoles of NADP + formed in the reaction (extinction coefficient of 0.00373 µM-1).

### Statistics

Analyses and graphs were made with GraphPad Prism version 8.0 (GraphPad Software, SD, CA, USA). The Shapiro-Wilk and Kolmogorov-Smirnov test was used to assess normality. Data were analyzed using a *student’s t-test* and *Welch’s t-test* are presented as mean ± standard error (SEM). A significance threshold of *p* < 0.05 was considered statistically significant.

## Results

To gain a deeper understanding of the cerebellar alterations induced by PCA, most of the experimental data were used to compare the mitochondrial network and pro-oxidant status across the molecular, Purkinje, and granular layers of the cerebellar cortex. We used a combination of three approaches to accomplish the structural and functional results of this study: (1) Ultrastructural analysis using electron microscopy to characterize mitochondrial presence and morphometry; (2) assessment of mitochondrial properties and pro-oxidant status utilizing fluorescent probes and biochemical techniques; (3) examination of mitochondrial dynamics through immunohistochemical and western blot experiments.

### Mitochondrial morphometry

**Fig. 1:**
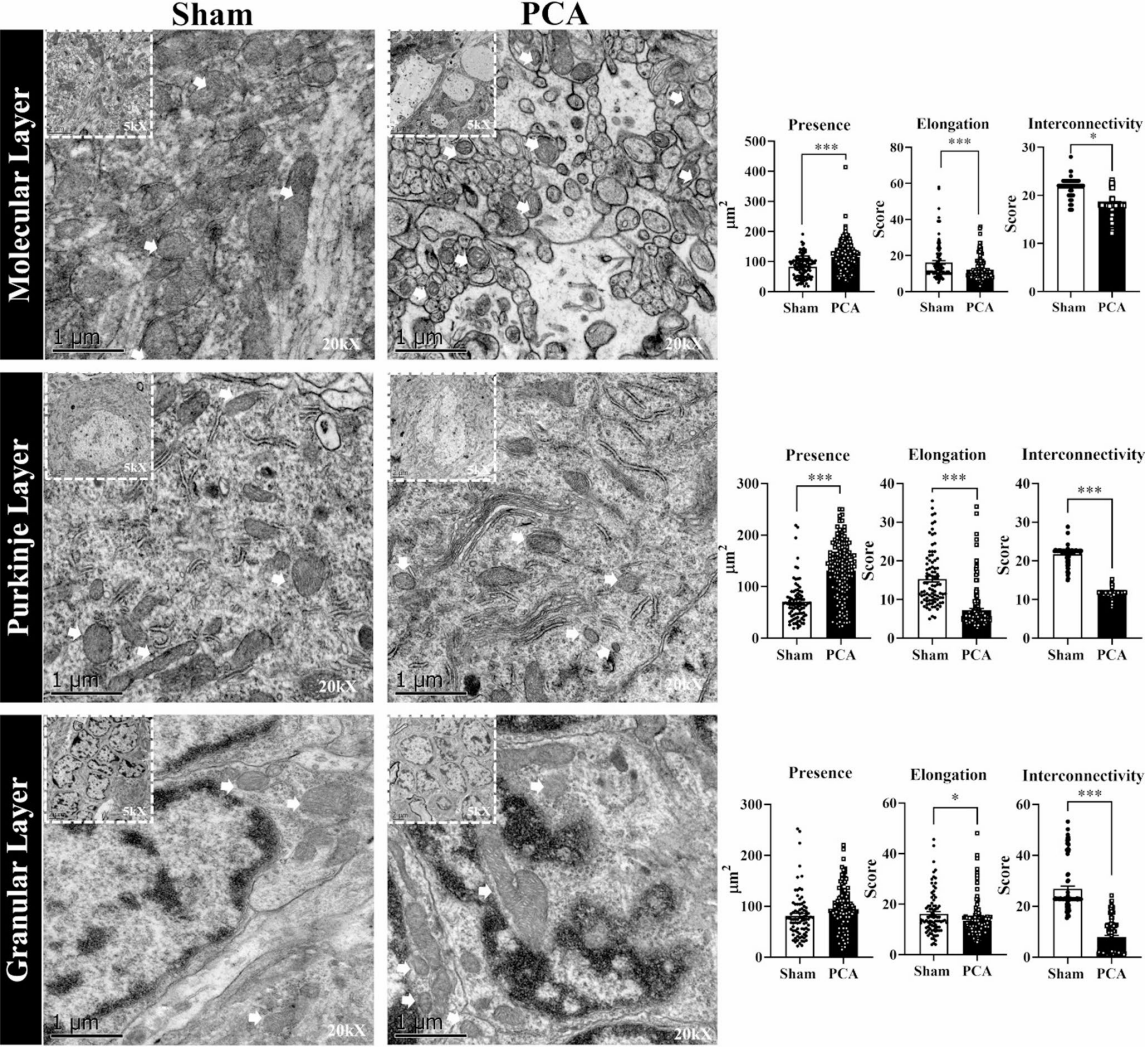
Morphometric analysis of the cerebellar cortex of PCA 13 rats by ultrastructural microscopy. Top: Higher-magnification of 14 electron microscopy in the molecular layer, white arrows show 15 mitochondrial network in Sham rats (left) and PCA rats (right), lower- 16 magnification inset. Middle: Micrographs of Sham and PCA rats of 17 Purkinje layer high magnification showing the mitochondrial network; 18 Bottom: representative micrographics of Granular layer in Sham rats 19 (left) and PCA rats (right), white arrowheads show the mitochondrial 20 network. Besides morphometric parameters: Mitochondrial presence, 21 Elongation, Interconnectivity of the mitochondrial network in each layer 22 were evaluated. Welch's t-test comparing Sham and PCA rats (*, p<0.01; 23 ***, p<0.0001) indicates significant differences between groups. 24 Representative images of 10 independent observations of mitochondrial 25 entities (~87 control and ~146 PCA)

PCA induced alterations in the quantity, area, and shape of mitochondria in the molecular, Purkinje, and granular layers of the cerebellar cortex (Fig. 1). Figures 1 presents representative electron micrographs at two magnifications, low magnification (5 kX) at insets for panoramic perspective, and higher magnification (20 kX) to highlight mitochondrial morphometry. PCA rats consistently exhibited a significant increase in mitochondrial presence, calculated based on the number and area of the mitochondria. Mitochondria increased by more than 90% in the molecular layer (*t = 9.9*,* SEM ± 6.0*,* p < 0.0001*), less than 50% in the Purkinje layer (t = 6.1, SEM ± 5.3, *p < 0.0001*) but we cannot detect a significant difference in the presence of mitochondria in the granular layer. PCA favored a reduction in mitochondrial elongation in all cerebellar cortex layers. Again, the effect was more significant in the mitochondria of the molecular *(t = 9.1*,* SEM ± 0.88*,* p < 0.0001)* and granular *(t = 2.3*,* SEM ± 1.1*,* p = 0.018)* layers, where mitochondrial elongation was reduced by nearly 50%. In the Purkinje layer, elongation was reduced by ~ 25% (*t = 3.5*,* SEM ± 1.1*,* p = 0.0005)*. Mitochondrial interconnectivity was also altered in the cerebellar cortex of PCA rats. This parameter was reduced in the three layers, with a similar pattern to the one observed with the mitochondrial elongation, indicating a more fragmented mitochondrial network in the cerebellar cortex layers of PCA rats.

### Mitochondrial membrane potential

**Fig. 2:**
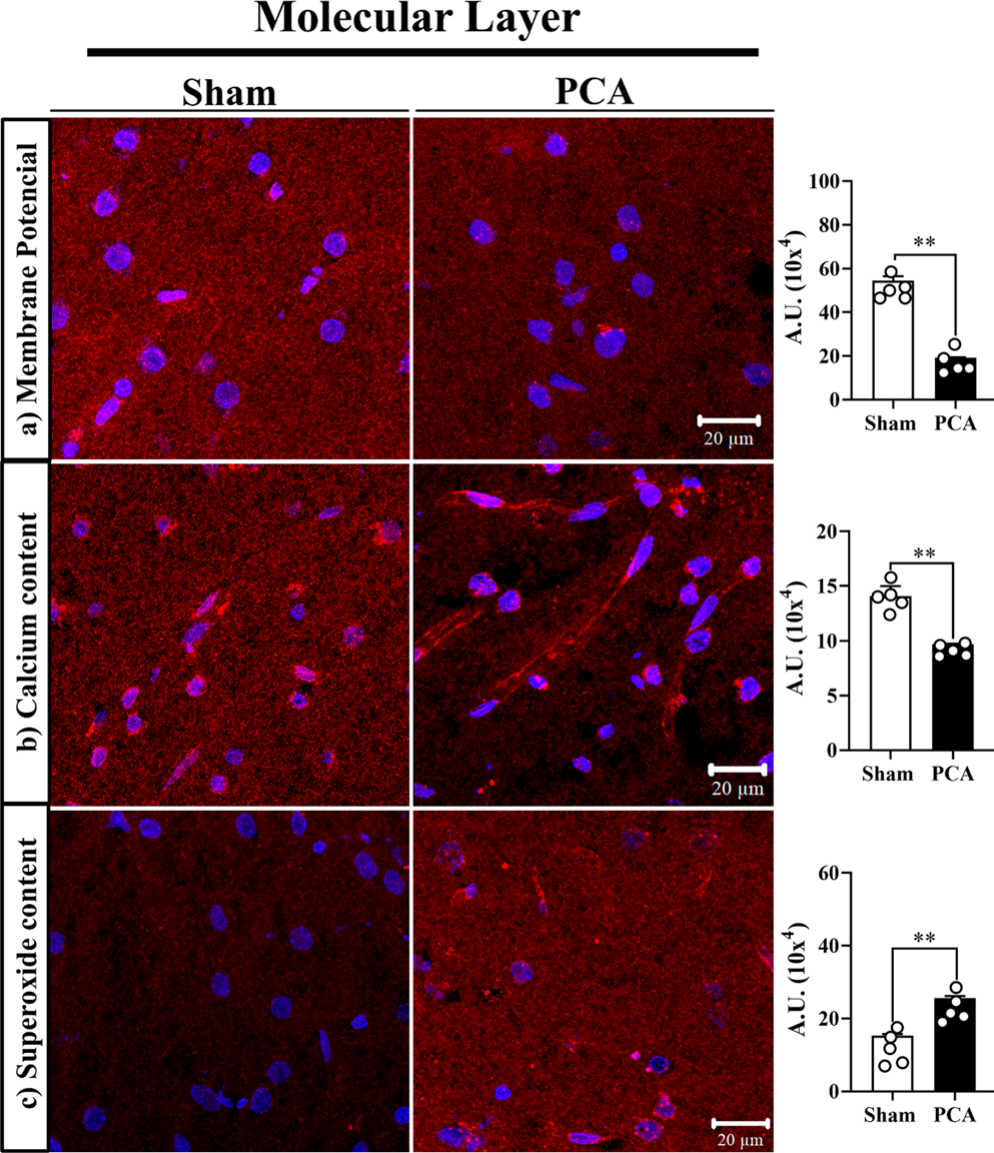
Mitochondrial biochemical parameters in the molecular 27 layer of PCA rats. Top: Mitochondrial membrane potential (ΔΨm) by 28 representative images of MitoTrackerRed in the molecular layer 29 corresponding to Sham rats (left) and PCA rats (right); graph shows the 30 fluorescence quantification. Middle: Mitochondrial calcium content in 31 the cerebellar cortex by representative images of DihydroRhod-2 32 fluorescent signal of the Molecular layer corresponding to Sham rats 33 (left) and PCA rats (right). Bottom: Representative imagens of MitoSOX ACCEPTED MANUSCRIPT Accepted manuscript 44 1 fluorescent signal are indicative of mitochondrial superoxide content in 2 the Molecular layer. Sham rats (left) and PCA rats (right). Graphs show 3 fluorescence quantification in each layer between groups; Student’s t- 4 test comparing Sham and PCA rats (**, p<0.001), n=5

Mitochondrial membrane potential (Δψ), a key factor in the bioenergetic process associated with oxidative phosphorylation, was measured by the MitoTracker Red fluorescent signal (Fig. 2, upper section panel a). Fluorescence values associated with MitoTracker Red (MTR) were normalized to the presence of mitochondria in each layer of the cerebellar cortex (Fig. 1). We consistently observed a significant reduction in the mitochondrial membrane potential across all cerebellar cortex layers, ranging from ~ 46% in the Purkinje layer (*t = 2.1*,* SEM ± 1.5*,* p = 0.0186*) to 67% in the molecular layer (*t = 5.3*,* SEM ± 2.0*,*p = 0.0061*) and significant reduction of ~ 50% in the granular layer (*t = 6.9*,* SEM ± 3.2*,*p < 0.0001*). The fluorescent signals and the corresponding graphic corresponding to the molecular layer are represented in Fig. 2, upper section to simplify the display of the results. Similar reduction was observed in Purkinje and granular layers (supplementary Fig. 1).

### Mitochondrial calcium content

Calcium plays a key role in the mitochondrial regulation of metabolic and electrical properties. To ensure and optimize the quantification of the mitochondrial Ca^2+^ content, we measured the fluorescent signal of dihydroRhod-2 normalized to the mitochondrial presence in our cerebellar system (Fig. 2, middle section panel b). The results showed a differential calcium content within the mitochondria of the cerebellar cortex of PCA rats: ~32% reduction in the molecular layer (*t = 4.7*,* SEM ± 0.87*,*p = 0.0050*). A different pattern was observer in the Purkinje layer (~ 26% increase) (t *= 8.5*,* SEM ± 0.83*,* p < 0.0001)*, and no change in the granular layer (supplementary Fig. 2). The fluorescent signals and the corresponding graphic corresponding to the molecular layer are represented in Fig. 2, middle section to simplify the display of the results.

### Mitochondrial superoxide

Mitochondrial ATP synthesis is intrinsically connected to the formation of the free radical anion superoxide (O_2_^·−^) in the electron transport chain. Superoxide production in the three cerebellar layers of the PCA rat cerebellum was measured with the mitochondrial probe MitoSOX (Fig. 2, lower section panel c). A ~ 65% increase in superoxide was detected in the molecular *(t = 3.9*,* SEM ± 0.57*,*p = 0.0090)* and Purkinje layers *(t = 4.0*,* SEM ± 0.62*,*p = 0.0134)*, while a remarkable ~ 140% increase was observed in the granular *layer (t = 4.4*,* SEM ± 0.2*,*p = 0.0055)*. The fluorescent signals and the corresponding graphic corresponding to the molecular layer are represented in Fig. 2, lower section to simplify the display of the results. Similar reduction was observed in Purkinje and granular layers (supplementary Fig. 3).

### Intracellular reactive oxygen species

**Fig. 3:**
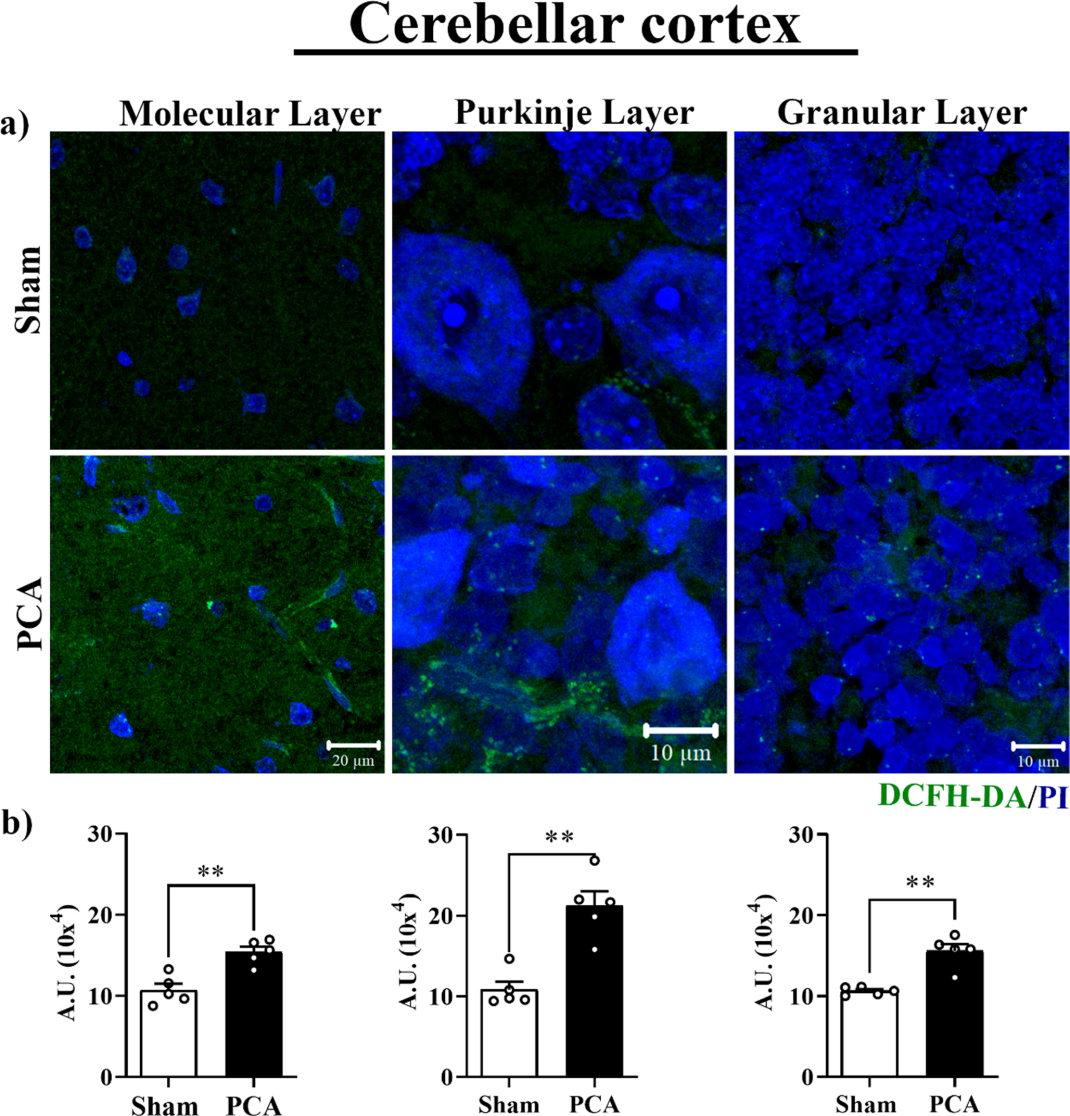
Intracellular ROS in the cerebellum cortex of PCA rats. 6 **a**) ROS intracellular content detection by DCFH-DA dye. Top: 7 representative image of Sham rats in the molecular, Purkinje, and 8 granular layers. Bottom: representative images of PCA rats in the 9 molecular, Purkinje, and granular layers. Graphs show fluorescence 10 quantification in each layer; Significant differences between groups were 11 calculated by student’s t-test, (**, p<0.001), n=5

Intracellular levels of ROS in PCA rats were measured using the fluorescent signal of the DCFA-DA across the three cerebellar cortex layers (Fig. 3, upper section and panel a). The surge in reactive oxygen intermediates was significant in the molecular layer ~ 95% *(t = 4.5*,* SEM ± 1.0*,*p = 0.0021)*, intermediate in the granular layer ~ 65% *(t = 5.4*,* SEM ± 0.90*,*p = 0.0038*, and moderate in the Purkinje layer ~ 48% *(t = 5.1*,* SEM ± 2.0*,*p = 0.0020).*

### Pro-oxidant and antioxidant reactions

**Fig. 4:**
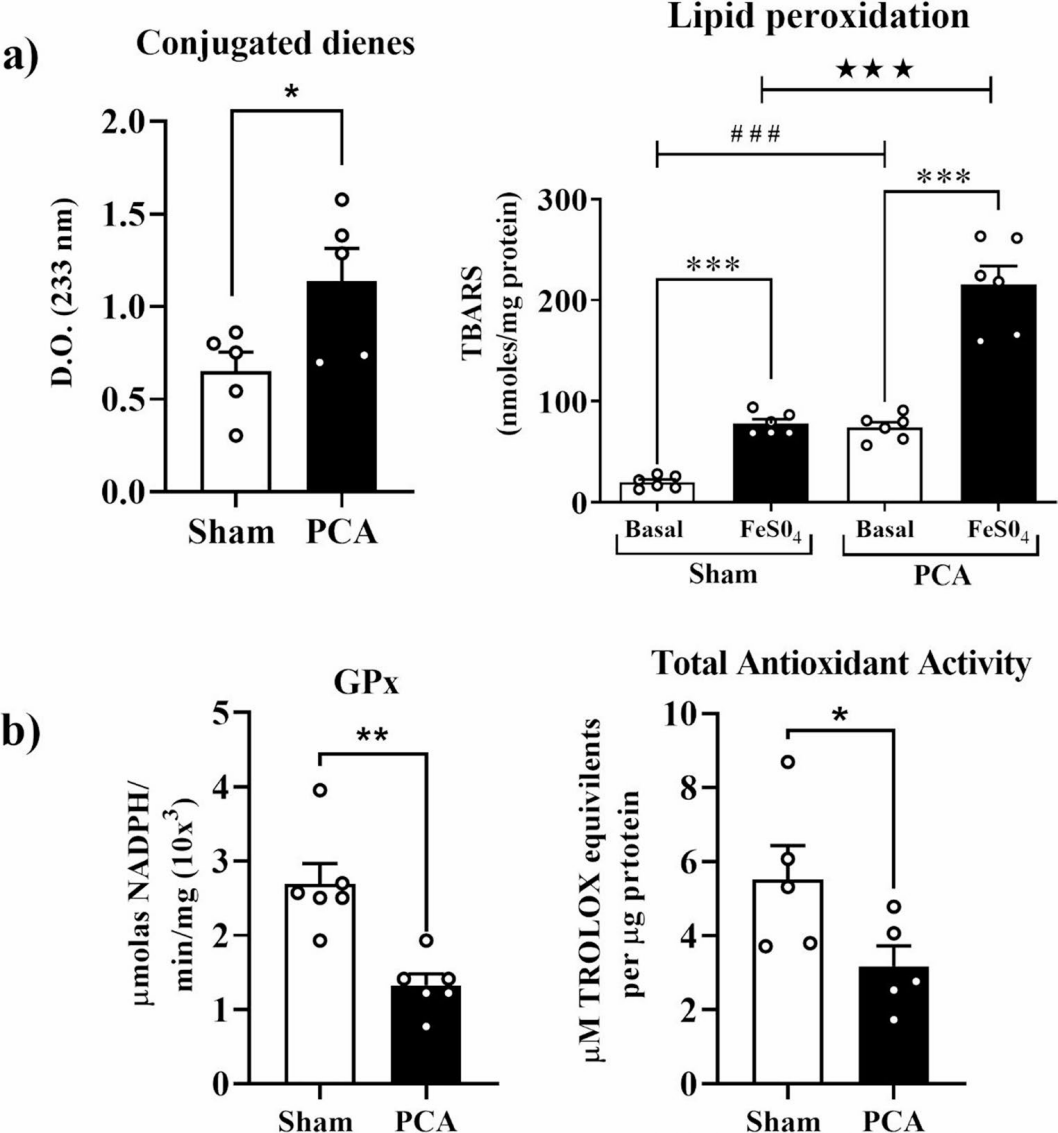
Pro-oxidant/antioxidant status in the cerebellum of PCA 13 rats. Parameters: **a**) left Graphs show conjugated dienes content. Right, 14 Lipid peroxidation showing by TBARS levels, depicting significant 15 differences between basal and maximum capacity of lipid peroxidation in 16 presence of FeSO4. **b**) Left, Glutathione peroxidase (GPx) activity. Right, 17 Total antioxidant capacity by CUPRAP test: Significance between groups 18 were calculated by t-test and Welch's t-test (*, p<0.01; ** p<0.001;★★★ 19 p<0.0001; *** p<0.0001; ### p<0.0001, n=6

The complex sequence of oxidative reactions in the cerebellar homogenate of PCA rats was characterized by measuring the lipoperoxidative activity using conjugated dienes and TBARS assays (Fig. 4a). In addition to the pro-oxidant markers, the total antioxidative capacity in and cerebellar GSH-per activity were also determined (Fig. 4b). Lipid peroxidation is a self-regenerative process that takes place in biological membranes. The atomic rearrangement of double bonds that generate conjugated dienes occurs during the initiation stage. Figure 4a left indicates that PCA promoted a significant increment in cerebellar conjugated dienes (∼77%; *t = 2.3*,* SEM ± 0.20*,* p = 0.049*). The TBARS assay was done in two modalities to characterize the propagative stage of lipid peroxidation (basal determination) and the maximum pro-oxidant capacity (supplemented with FeSO_4_). The cerebellar homogenate in PCA rats showed a significant increase (*t = 7.4*,* SEM ± 19.05*,* p = 0.0004)* in both TBARS assays with a more pronounced elevation in the basal determination *(7-fold increase*,* t = 7.7*,* SEM ± 6.9*,* p = 0.0006)* than in the presence of Fe^2+^ (*3-fold increase*,* t = 8.5*,* SEM ± 16.01*,* p = 0.0004)* (Fig. 4a right). It can be observed in panel b that the enhanced cerebellar pro-oxidizing response was corroborated by a significant decline in total antioxidant activity, as indicated by the CUPRAC test (∼46%; *t = 3.5*,* SEM ± 0.65*,* p = 0.023)* in PCA rats (Fig. 4b, right), as well as a reduction in cerebellar GSH-per activity (~ 49%; *t = 6.5*,* SEM ± 0.20*,* p* = 0.0013) (Fig. 4b, left).

**Fig. 5:**
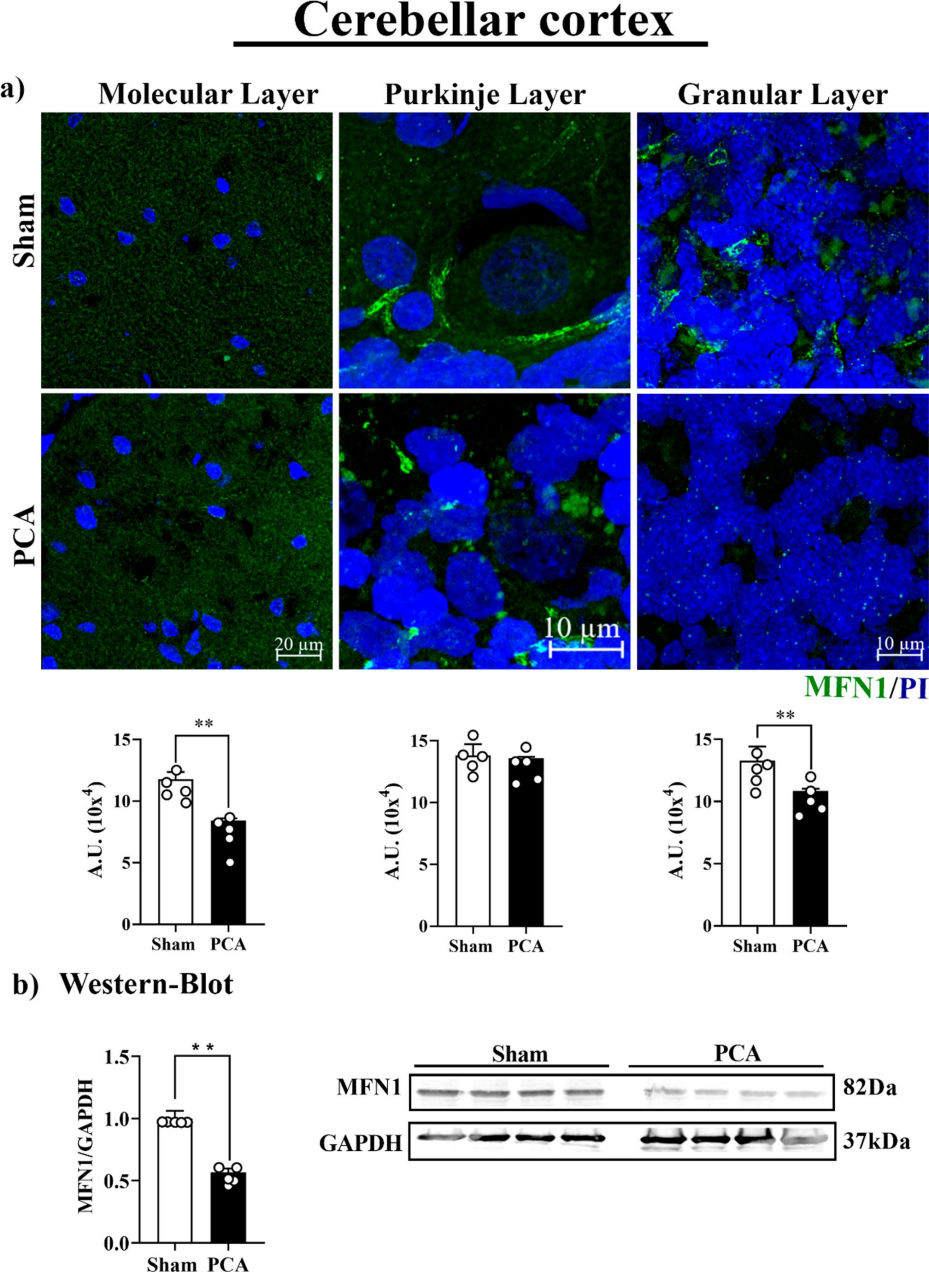
Mitofusin 1 (MFN1) protein evaluation in the cerebellar 21 cortex of PCA rats. Representative images of the molecular, Purkinje, 22 and granular layers of sham rats (top) and PCA rats (bottom). **a**) Graphs 23 show the quantification of the fluorescent signal in each cerebellar layer. 24 **b**) Representative Western blot images and densitometric analysis of 25 MFN1 protein expression. Protein levels were normalized to GAPDH 26 expression. Data are reported as mean ± SEM. Significance was 27 calculated with student’s t-test (**, p<0.001), n=5

**Fig. 6:**
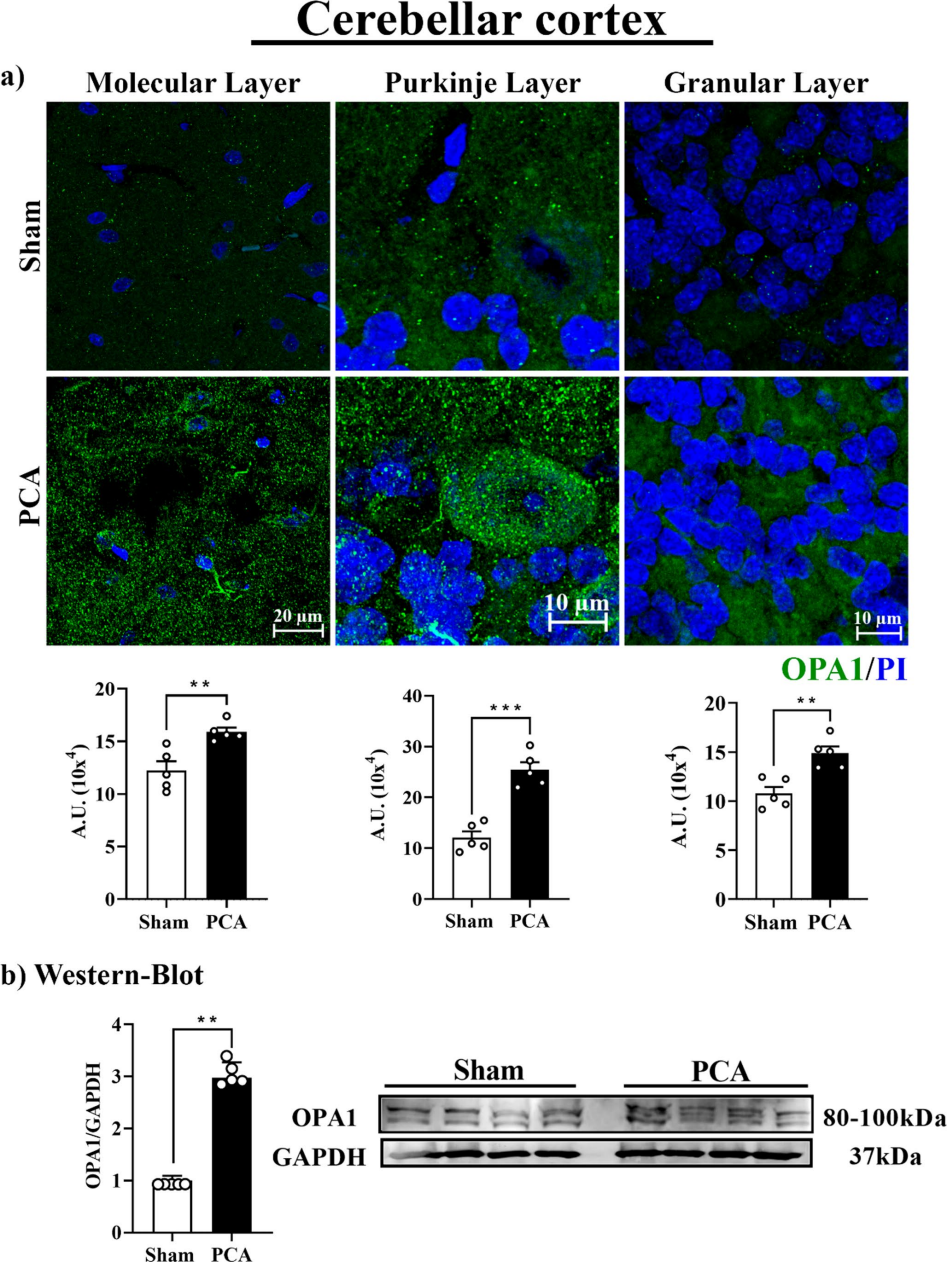
Optic atrophy 1 (OPA1) protein evaluation in the 29 cerebellar cortex of PCA rats. Representative images of the molecular, 30 Purkinje, and granular layers of sham rats (top) and PCA rats (bottom). 31 **a**) Graphs show the quantification of the fluorescent signal in each 32 cerebellar layer. **b**) Representative Western-Blot images and 33 densitometric analysis of OPA1 protein expression. Protein levels were 34 normalized to GAPDH expression. Data are reported as mean ± SEM. 35 Significance was calculated with student’s t-test (**, p<0.001; ***, 36 p<0.0001), n=5

To further explore the condition of the mitochondrial network in the PCA rat cerebellum, fusion markers (MNF1, MNF2 and OPA1) were evaluated through immunohistochemical and Western blot analyses (Figs. 5 and 6, supplementary Fig. 4). The data were also normalized to the presence of mitochondria in each cerebellar cortex layer. It can be observed in Fig. 5 that MFN1 levels were clearly reduced (~ 40%; *t = 11.91*,* SEM ± 0.031*,* p = 0.0013*) in the PCA rat cerebellum, as evidenced by the Western blot analysis (Fig. 5b). The reduction of MFN1 was more accentuated in the molecular *(t = 3.4*,* SEM ± 0.10*,* p = 0.0036)* and granular layers *(t = 4.0*,* SEM ± 0.82*,* p = 0.0042)* (~ 34 and 20%, respectively), but the levels of this factor remained unchanged in the Purkinje neurons (Fig. 5a). Alongside the lower levels of MFN1, it was noted that this mitofusin formed aggregates, especially within the Purkinje layer of sham. MFN2 showed a *reduction *(~ 40%; *t = 5.5*,* SEM ± 0.79*,* p = 0.0015)* in the molecular layer whereas any change was detected in the Purkinje and granular layers. Wester-blotting show a significant reduction *(t = 8.0*,* SEM ± 0.06*,* p = 0.0040)* (supplementary Fig. 4).

In contrast, western blot analysis demonstrated a substantial increase in cerebellar OPA1 in the PCA group *(*~ 192%; *t = 6.6*,* SEM ± 0.29*,* p = 0.0070) (*Fig. 6). The OPA1 signal also showed that PCA cerebella expressed alternative isoforms of OPA1 not detected in the sham rats, mainly at medium molecular weight. OPA1 enhancement was also notable in the Purkinje layer (~ 117%; *t = 6.8*,* SEM ± 1.93*,* p = 0.0002*) and moderate in the granular and molecular layers *(~ 32%; t = 4.25*,* SEM ± 0.96*,* p = 0.0028* and *~ 38%; t = 3.8*,* SEM ± 0.94*,* p = 0.0092*, respectively).

### Mitochondrial fission markers

**Fig. 7:**
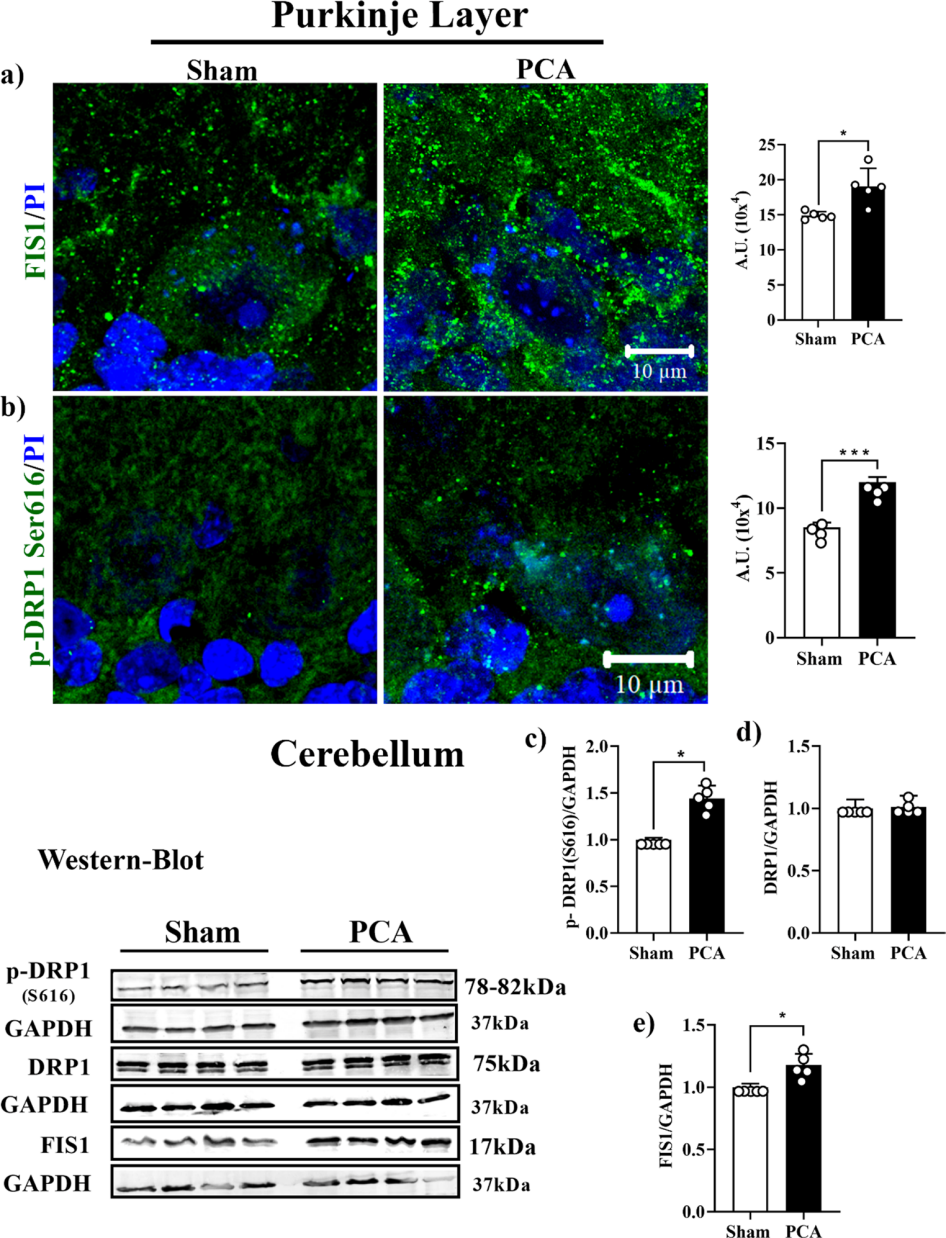
Mitochondrial Fission markers by protein evaluation in 38 the cerebellar cortex of PCA rats. **a**) Mitochondrial Fission protein 1 39 (FIS1) presence in Purkinje layer Representative images of Sham rats 40 (left) and PCA rats (right). **b**) Dynamin-related protein 1 (DRP1) ACCEPTED MANUSCRIPT Accepted manuscript 45 1 Phosphorylated Serine 616 (Ser616). Representative images of Purkinje 2 layer in Sham rats (left) and PCA rats (right). Graphs show the 3 quantification of the fluorescent signal in each cerebellar layer. **c**) 4 Western-Blot images and densitometric analysis of **d**) p-DRP1 Ser616 5 protein expression. **e**) total DRP1, f) FIS1 protein expression. Protein 6 levels were normalized to GAPDH expression. Data are reported as mean 7 ± SEM. Significance was calculated with student’s t-test (*, p<0.01; ***, 8 p<0.0001)

Two factors associated with mitochondrial fragmentation, FIS1 and DRP1 phosphorylated at serine 616 were evaluated in the cerebellum of PCA rats (Fig. 7). The presence of FIS1 (Fig. 7a) and p-DRP1-Ser616 (Fig. 7b) were significantly enhanced in the Purkinje layer *(*∼*33%; t = 3.5*,* SEM ± 1.17*,* p = 0.0208* and ∼*62%; t = 6.2*,* SEM ± 0.55*,* p = 0.0008*, respectively). The Western blot signals in the complete cerebellum (Fig. 7c) were also enhanced for the two proteins *(*∼*20%*,* t = 5.1*,* SEM ± 0.089*,* p = 0.0139*,* ~ 60%; (t = 3.2*,* SEM ± 0.13*,* p = 0.0186)*, respectively) (panels d and F). However, total DRP1 protein did not show significant change (Fig. 7e).

The presence of FIS1 was also increased in the molecular and granular layers in the PCA rats (supplementary Figs. 5). Fis1 enhanced ~ 38% in both, the molecular and granular layers *(t = 3.14*,* SEM ± 1.6*,* p = 0.01 and t = 4.0*,* SEM ± 1.53*,* p = 0.0071*) respectively. No significant changes were detected in the presence of p-DRP1 Ser616 in the molecular and granular layers of PCA rats (supplementary Fig. 6).

## Discussion

The complexity of the cerebellar cortex’s diverse cellular types, synaptic connections, and physiological and transcriptional properties have been updated in specialized reports (Hull and Regehr 2022; Kozareva et al. 2021). These reports have also shown that cellular regionalization is present when posterior versus anterior cerebellar lobules are also considered (Kozareva et al. 2021). Therefore, our experimental approach was pragmatic; we analyzed the cerebellar cortex, focusing on ultrastructural features, fluorescent probe signals, and the presence of mitochondrial markers in the molecular, Purkinje, and granular layers.

Years ago, it was believed that HE episodes associated with liver failure were essentially a metabolic problem caused by high ammonia levels (the sum of ionized NH_4_^+^ and non-ionic NH_3_) and astrocyte dysfunction (Szerb and Butterworth 1992). However, holistic and contemporary approaches view HE as a complex expression of an altered function of the neurogliovascular unit. According to these approaches, neuronal metabolic and cellular events that accompany this pathology arise from the combination of anomalous intrinsic brain factors (derived from neurons, astrocytes, microglia, endothelial cells, etc.) along with parallel dysfunction in peripheral organs. Hence, HE is described as a global impairment in cellular communication within the brain, driven by the concurrence of hyperammonemic, pro-inflammatory, and pro-oxidant states (Claeys et al. 2021).

The present manuscript aimed to further characterize the multiple cerebellar abnormalities observed in male rats after 13 weeks with PCA (López-Cervantes et al. 2021). This study focused on exploring mitochondrial, pro-oxidant, and ultrastructural alterations specifically in the granular, Purkinje, and molecular layers of the cerebellar cortex of operated rats. It is relevant to point out that 13 weeks of PCA is recognized as an advanced state of hepatic hypofunctioning (Vázquez-Martínez et al. 2019) that is accompanied by altered cerebellar coordination, loss of Purkinje cells, the presence of inflammatory markers, and changes in cerebellar metabolite levels (López-Cervantes et al. 2021). However, Cavanagh et al. considered 13 weeks of PCA as a period during which spongiform vacuolization remained active (Cavanagh et al. 1972).

PCA is a distinctive model of experimental portal-systemic encephalopathy. In 1983, Pilbeam and colleagues noted that pathological responses in the PCA and CCl_4_ models were clearly different. CCl_4_-induced cirrhosis with terminal coma produces diffuse gliosis, necrotic events, and type II astrocytes, but not cerebellar vacuolization. These changes are accompanied by severe behavioral deficits and high levels of hyperammonemia. In contrast, PCA is associated with minor neurological symptoms, astrocytic nuclear swelling, and swelling of the astrocytic end-foot processes. However, spongiform degeneration is evident in the molecular layer of the cerebellum. It is also contrasting that PCA promotes only a discreet elevation in circulating ammonium (Pilbeam et al. 1983).

Consistent with our findings, a recent publication linking episodes of HE with thioacetamide and bile duct ligation, also concluded that mitochondrial and oxidative stress were involved in pathogenic mechanisms, especially in mouse GABAergic neurons of the substantia nigra pars reticulata (Bai et al. 2023). Interestingly, these authors reverted the mitochondrial dysfunction in this model by overexpressing uncoupling protein 2 (UCP2) and inhibiting glutamate decarboxylase 2 activity (Bai et al. 2023). The protective action of UCP2 strongly suggests a mitochondrial role in the oxidative damage observed in these models of hepatic failure (Hass and Barnstable 2021).

### Pro-oxidant status and mitochondrial dynamics

The fluorescent and biochemical results indicate a redox imbalance that favors an increased pro-oxidant condition in the PCA cerebellum. It should be considered that after 13 weeks post-surgery, this oxidative stress correlates with an active pro-inflammatory state and an active process of cellular loss (López-Cervantes et al. 2021). Indeed, the enhanced pro-oxidant status in PCA rats may be related to the mitochondrial alterations observed in this study, as the cellular systems in the cerebellum have remained functional by adapting to ongoing damage after 13 weeks of systemic metabolic distress due to reduced hepatic function.

The physiology and structure of the mitochondrial network are fundamental to cellular bioenergetics and the equilibrium between anabolic and catabolic reactions. Mitochondria respond to various metabolic demands and stressful events and are capable of extensive component exchange, morphologically adapting, coordinating with other organelles, and acting as important factors in intercellular communication. Indeed, pathological situations may compromise these capabilities (Chen et al. 2023). Our data in the analyzed images from electron microscopy showed that in the three layers of the cerebellar cortex in PCA rats, mitochondria exhibited higher fragmentation, by fluorescent markers it was also observed reduced membrane potential, and an increase in superoxide anion. In addition, we confirmed that the pro-oxidant status was present in the whole cerebellum. Several reports indicate that stressors, such as ROS and Ca^2+^, induce fragmentation of the mitochondrial network in the context of degenerative disease progression (Angelova and Abramov 2018; Annesley and Fisher 2019; Chen et al. 2023; Napolitano et al. 2021). Interestingly, only some of the markers of mitochondrial fusion-fission corresponded with the fragmented network detected by electron microscopy: reduction in MFN1 and enhancement of FIS1 and phosphorylated DRP-Ser616. In this context, it is remarkable that the evident increase of OPA1 in the cerebellar cortex of PCA rats. It is worth noting that the Western blot experiment for OPA1 (Fig. 5) showed the appearance of a short isoform of this protein (S-OPA1). Cellular stress activates metalloprotease OMA1, leading to L-OPA1 cleavage and the formation of S-OPA1. Research shows that higher levels of S-OPA1 have been shown to enhance cell survival under adverse conditions (Giacomello et al. 2020). Furthermore, S-OPA1 has been proposed to play a role in the plasticity of mitochondrial cristae, which is essential for the functional assembly of respiratory complexes and supercomplexes (Cogliati et al. 2013).

OMM proteins, such as MFN1 and 2, orchestrate mitochondrial fusion. MFN1 has been shown to participate primarily in mitochondrial fusion by cooperating with OPA1, while MFN2 also tethers mitochondria to the endoplasmic reticulum (ER). Molecular ablation of MFN2 in mouse embryonic fibroblasts and HeLa cells alters ER morphology and weakens ER-mitochondria interactions, which reduces the efficiency of mitochondrial Ca^2+^ uptake (Giacomello et al. 2020; Gilkerson et al. 2021). Our data showed an increase of fragmented mitochondria in the cerebellar cortex (Figs. 1). This pattern is consistent with the reduced presence of MFN1 in the molecular and granular layers, as well as the lower expression of MFN1 in the Western blot experiment (Fig. 5). So far, we do not have elements to characterize the interaction between mitochondria and the ER. As it was said, the clear enhanced presence of OPA1 could be related not to its fusion activity but to the capacity to adapt mitochondrial cristae plasticity under stressful conditions (Giacomello et al. 2020).

The fragmented mitochondria detected in the cortex layers were congruent with the generalized increase in FIS1 (Fig. 7 and supplementary Fig. 5). This result also indicates that mitochondrial fission in the PCA cerebellum is more associated with “pathological” mitochondrial fragmentation than with healthy physiological mitochondrial division (Mormina et al. 2017).

The fragmented mitochondrial network and the imbalance of fission and fusion processes have been associated with mitochondrial distress (Knott et al. 2008). Our results are consistent with higher levels of FIS1 and phosphorylation of DRP1 at Ser616 as well as reduced MFN1. This combination is consistent with the fissioned mitochondria detected by electron microscopy. In addition to these mitochondrial alterations, a reduced mitochondrial membrane potential and increased oxidative stress are characteristic of the pathological consequences of PCA after 13 weeks (Mormina et al. 2017).

### The cerebellum as a metabolic target

The present study and our previous report (López-Cervantes et al. 2021) focus on the cerebellar alterations detected in PCA rats after 13 weeks after microsurgery. The initial observation that motivated this interest was the exclusive presence of spongiform vacuolization in the molecular layer of the cerebellum in treated animals (Cavanagh et al. 1972; López-Cervantes et al. 2021). The cerebellum is susceptible to pathological and toxic conditions, as well as to genetic syndromes that specifically impact its structural and functional characteristics. For example, it has been reported that patients with multiple sclerosis, Parkinson’s disease, and various forms of ataxia present conspicuous cerebellar abnormalities (Mormina et al. 2017). Chronic administration of valproic acid results in distinctive functional and structural alterations in the cerebellum, causing symptoms similar to those of HE (Sobaniec-Lotowska and Sobaniec 1996). In patients with pre-senile and senile dementia of the Alzheimer’s type, numerous and large amyloid deposits were observed, especially in the molecular layer of the cerebellum (Braak et al. 1989). Regarding spongiform degeneration, a similar vacuolization in the molecular layer is a feature of Cavanagh disease, which is a genetic leukodystrophy related to the mutated aspartoacylase oligodendroglial gene. Patients with this neurodegenerative disease present high levels of N-acetylaspartate, reduced acetyl coenzyme A, and myelinating capacity (Wei et al. 2022). Spongiform degeneration is also detected in mitochondrial syndromes, such as Leigh disease (Schubert and Vilarinho 2020) and Kearns-Sayre syndrome (Tanji et al. 1999). Spongiform damage associated with prion protein deposits in cases of variant Creutzfeldt-Jakob disease was also detected in the cerebral cortex, hippocampus, dentate gyrus, and cerebellar molecular layer. An important proportion of florid-type prion protein plaques was observed, especially in the cerebellum (Armstrong et al. (Armstrong, et al., 2002)). Vacuolation damage has been reported with microbial factors, such as the exotoxin from the A strain of *Mycoplasma neurolyticum* (Thomas et al. 1966) and parvovirus infection (Schaudien et al. 2010). A Chinese report indicated that a form of a spongiform leucoencephalopathy was detected among heroin pyrolysate abusers (Lu et al. 2001).

## Limitations

While our study has provided valuable insights into the subcellular and biochemical alterations in the cerebellar cortex of PCA rats, there are several possibilities for further investigation. More research is warranted to unravel the underlying molecular and cellular mechanisms linking the spongiform vacuolization detected only in the molecular layer and the metabolic adaptations associated with the hyperammonemic conditions accompanying liver hypofunction. Longitudinal studies conducted before and after the 13 weeks of PCA after microsurgery are also needed to assess the onset and development of the observed differential ultrastructural and mitochondrial changes over time in the three cerebellar cortex layers.

There are several limitations to consider in our study that may impact the interpretation and perspectives of our findings. Our study did not investigate sex differences in the cerebellar damage. We focused only on male offspring, but we recognize the significance of studying females also. Furthermore, emphasize that we limited our experimental protocol to describe the cerebellar alterations associated with PCA. Indeed, it would be pertinent to extend the pathological characterization to other brain areas that are susceptible to spongiform changes in different models of cerebral damage, such as mitochondrial syndromes and prionic diseases. In addition, it would be relevant to consider cerebral regions that are documented to show cellular modifications such as type II astrocytes during PCA surgeries.

A notable limitation of our study is the lack of experiments to study oximetry in mitochondrial preparations. The aim of our protocol was to characterize metabolic changes in the three layers of the cerebellar cortex, which prevented that purpose. However, the possibility of assaying a mitochondrial preparation of whole cerebellar tissue could be interesting to pursue.

## Conclusion

In summary, after 13 weeks of PCA, our data shows active oxidative stress in the cerebellar cortex, accompanied by a fragmented mitochondrial network. However, the metabolic stress related to high levels of ammonia and the pro-inflammatory conditions differentially affected the three layers of the cerebellar cortex: spongiform vacuoles were exclusively present in the molecular layer, a greater proportion of Purkinje neurons was lost, and the mitochondria cristae exhibited more significant alterations in the molecular layer. Hence, the cellular alterations observed in the cerebellar cortex are representative of a tissue experiencing an advanced state of metabolic stress with important detrimental effects, while at the same time implementing significant adaptations to survive and function under adverse conditions.

## Supplementary Information

Below is the link to the electronic supplementary material.

Supplementary Material 1 (PDF 1,353 KB)

Supplementary Material 2 (PDF 437KB)

## Data Availability

No datasets were generated or analysed during the current study.
