# Supplementary material for: Portacaval anastomosis promotes fragmentation of mitochondrial network in the cerebellum of male rats
Source: Metab Brain Dis. 2025 Sep 24;40(7):274. doi: 10.1007/s11011-025-01705-8 (PMC12460505; doi:10.1007/s11011-025-01705-8)
Supplement: Supplementary file 1 — Supplementary Material 1 (PDF 1,353 KB) [file 11011_2025_1705_MOESM1_ESM.pdf]

OPA1

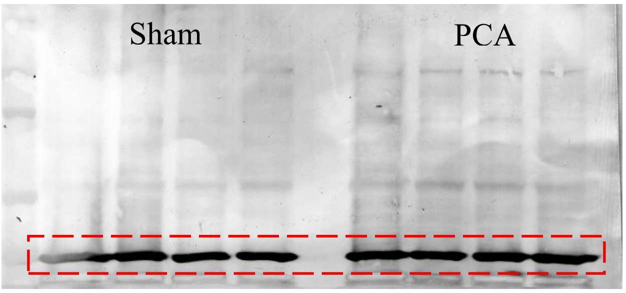

Western blotting of cerebellum, 7.5 % SDS-PAGE gel. GAPDH (37kDa)

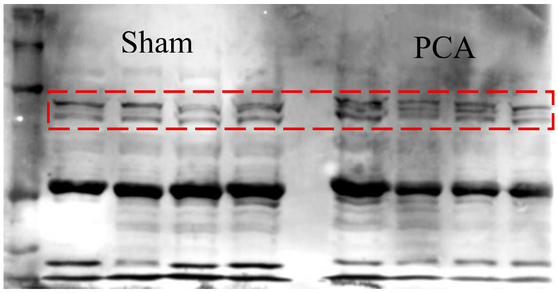

Western blotting of cerebellum, 7.5 % SDS-PAGE gel. OPA1 (80-100kDa)

MNF1

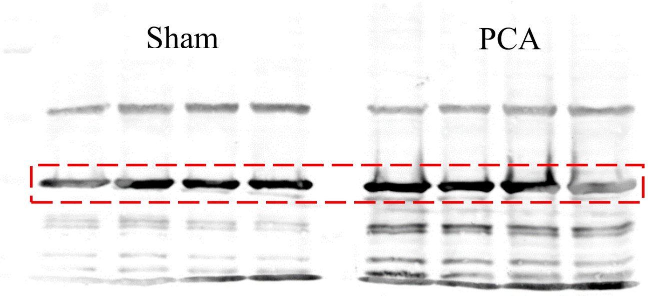

Western blotting of cerebellum, 10 % SDS-PAGE gel. GAPDH (37kDa)

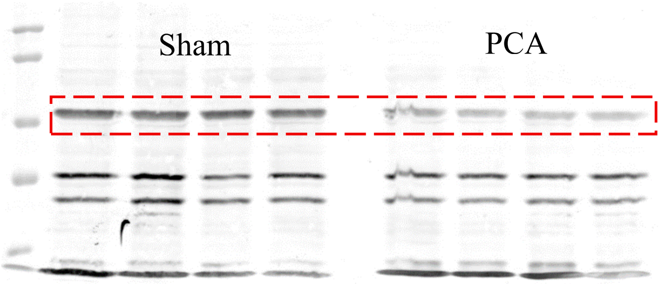

Western blotting of cerebellum, 10 % SDS-PAGE gel. MNF1 (82kDa)

MNF2

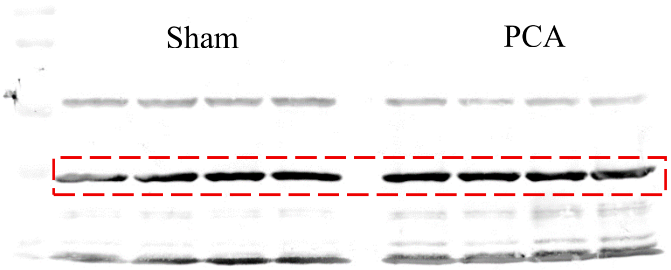

Western blotting of cerebellum, 10 % SDS-PAGE gel. GAPDH (37kDa)

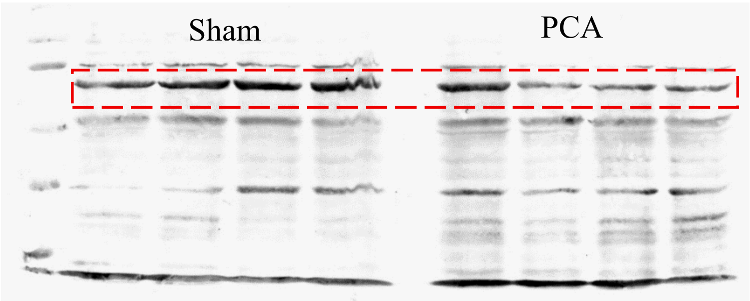

Western blotting of cerebellum, 10 % SDS-PAGE gel. MNF2 (80kDa)

FIS1

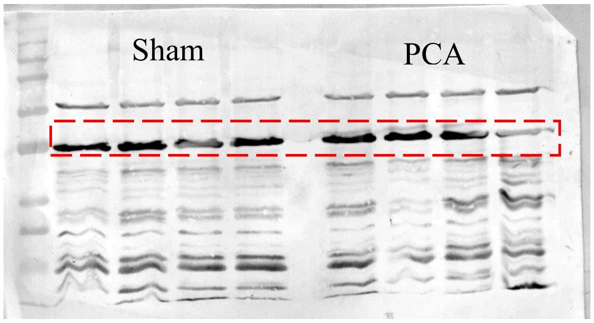

Western blotting of cerebellum, 15 % SDS-PAGE gel. GAPDH (37kDa)

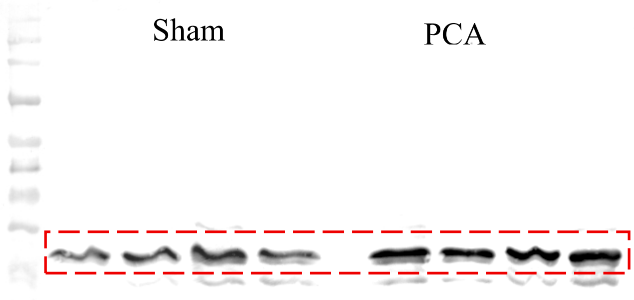

Western blotting of cerebellum, 15 % SDS-PAGE gel. FIS1 (17kDa)

DRP1

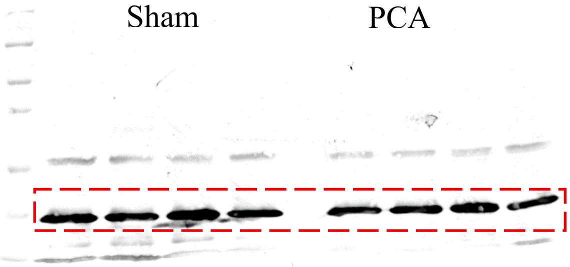

Western blotting of cerebellum, 10 % SDS-PAGE gel. GAPDH (37kDa)

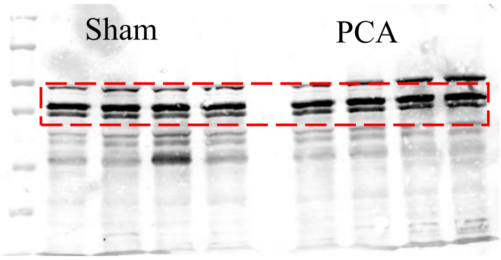

Western blotting of cerebellum, 10% SDS-PAGE gel. DRP1 (75kDa)

p-DRP1 Ser616

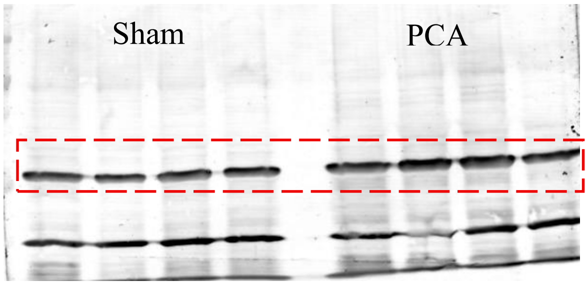

GAPDH Western blotting of cerebellum, 10 % SDS-PAGE gel. GAPDH (37kDa)

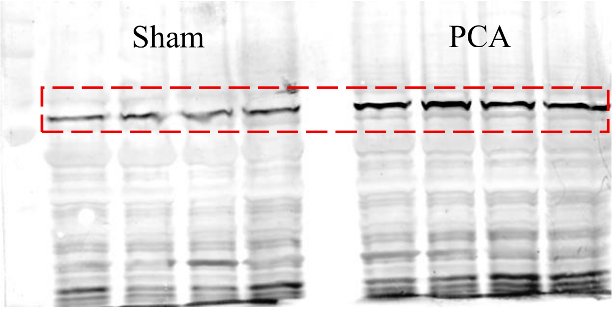

Western blotting of cerebellum, 10 % SDS-PAGE gel. p-DRP1 Ser616 (78-82 kDa)
