# Supplementary material for: Portacaval anastomosis promotes fragmentation of mitochondrial network in the cerebellum of male rats
Source: Metab Brain Dis. 2025 Sep 24;40(7):274. doi: 10.1007/s11011-025-01705-8 (PMC12460505; doi:10.1007/s11011-025-01705-8)
Supplement: Supplementary file 2 — Supplementary Material 2 (PDF 437KB) [file 11011_2025_1705_MOESM2_ESM.pdf]

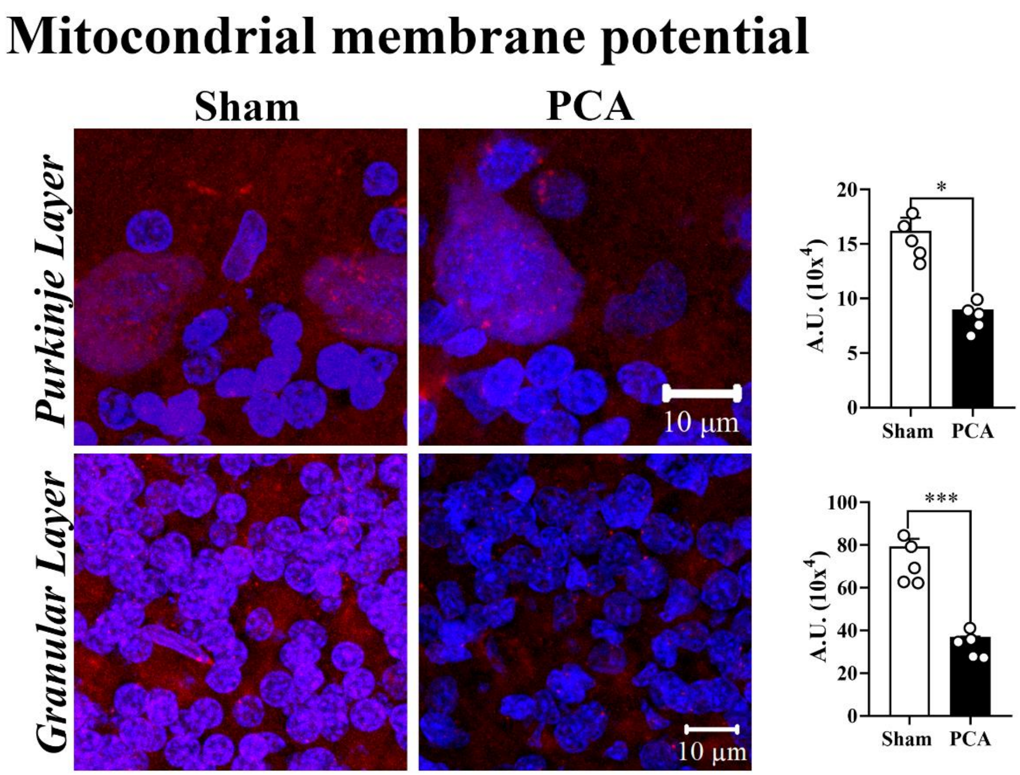

SUPPLEMENTARY FIGURE 1. **Mitochondrial membrane potential (ΔΨm) Purkinje and granular layers of PCA rats**. Top: Representative images of Purkinje layer with MitoTrackerRed corresponding to Sham rats (left) and PCA rats (right); graph shows the fluorescence quantification. Bottom: Representative imagens of granular layer with MitoTrackerRed, Sham rats (left) and PCA rats (right). Graphs show fluorescence quantification in each layer between groups; Student's *t-test* comparing Sham and PCA rats (*, $p<0.05$; ***, $p<0.0001$), n=5.

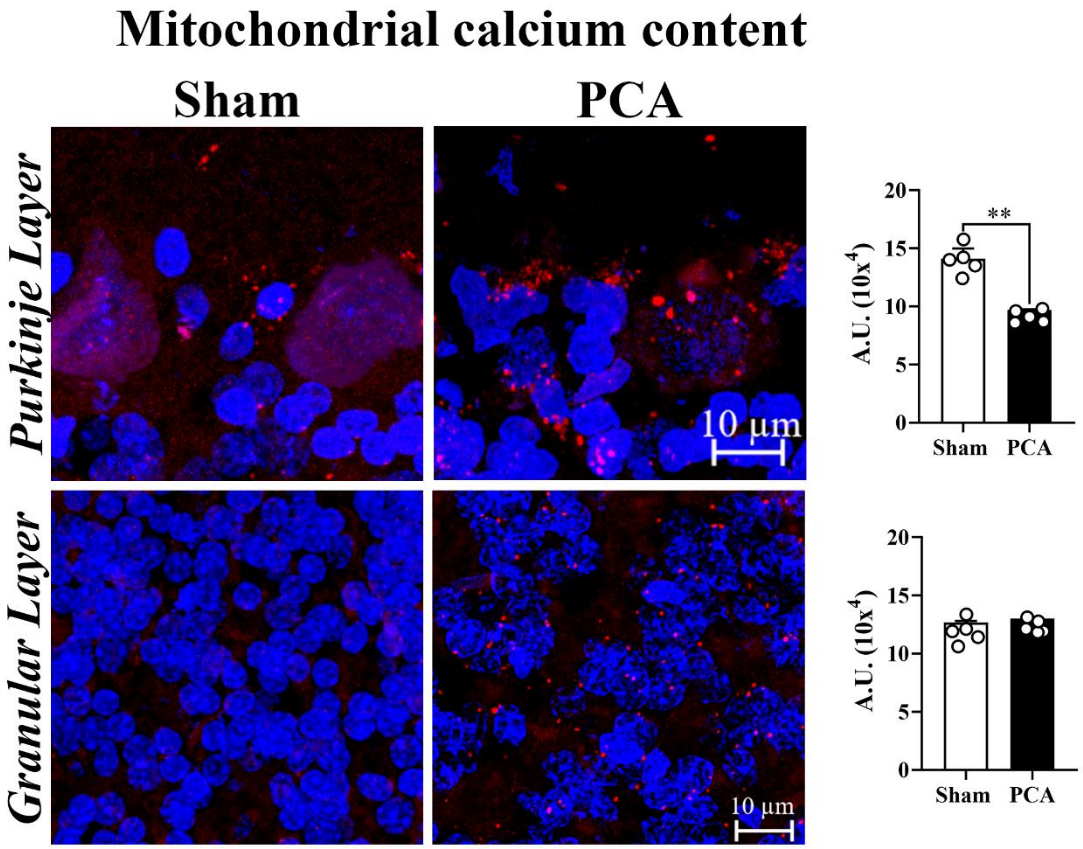

SUPPLEMENTARY FIGURE 2. **Mitochondrial calcium content by DihydroRhod-2 in Purkinje and granular layers of PCA rats**. Top: Representative images of Purkinje layer with DihydroRhod-2 corresponding to Sham rats (left) and PCA rats (right); graph shows the fluorescence quantification. Bottom: Representative imagens of granular layer with DihydroRhod-2, Sham rats (left) and PCA rats (right). Graphs show fluorescence quantification in each layer between groups; Student's *t-test* comparing Sham and PCA rats (**, $p<0.001$), n=5.

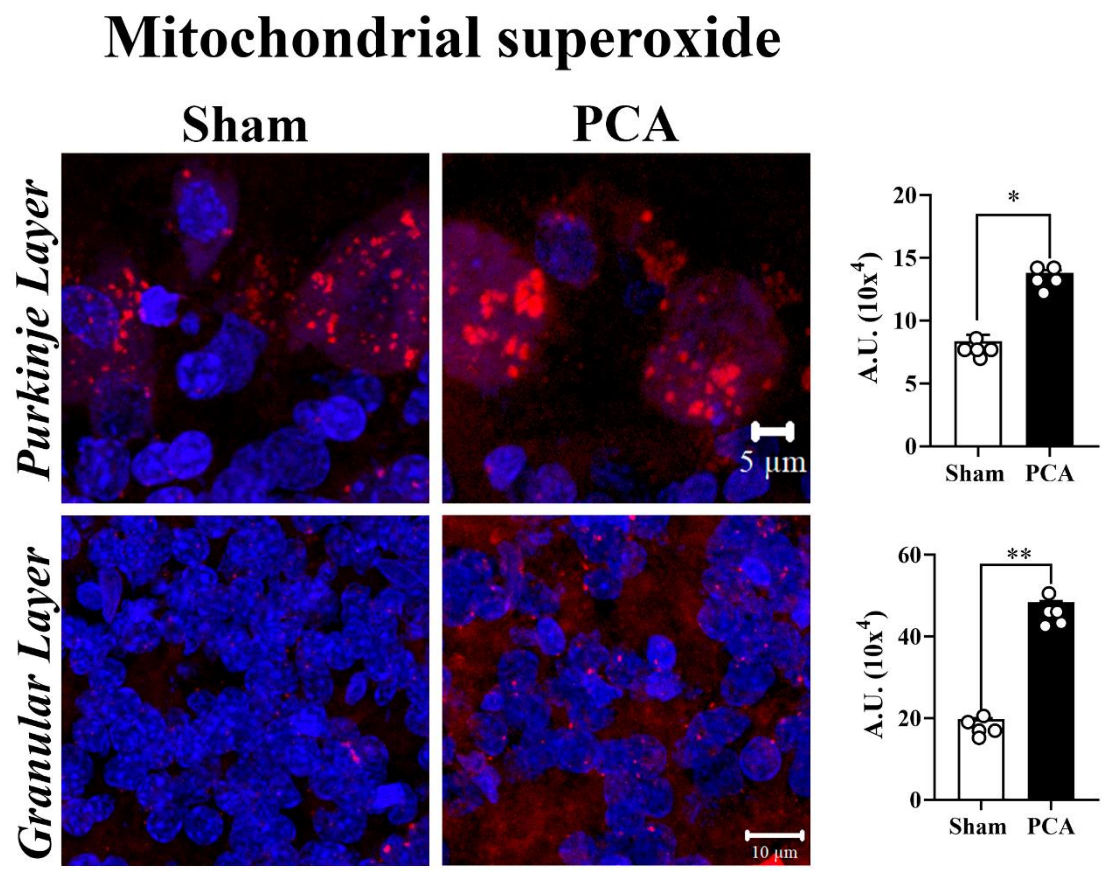

SUPPLEMENTARY FIGURE 3. **Mitochondrial superoxide content by MitoSOX in Purkinje and granular layers of PCA rats**. Top: Representative images of Purkinje layer with MitoSOX corresponding to Sham rats (left) and PCA rats (right); graph shows the fluorescence quantification. Bottom: Representative imagens of granular layer with MitoSOX, Sham rats (left) and PCA rats (right). Graphs show fluorescence quantification in each layer between groups; Student's *t-test* comparing Sham and PCA rats (*p<0.01; ** p<0.001), n=5.

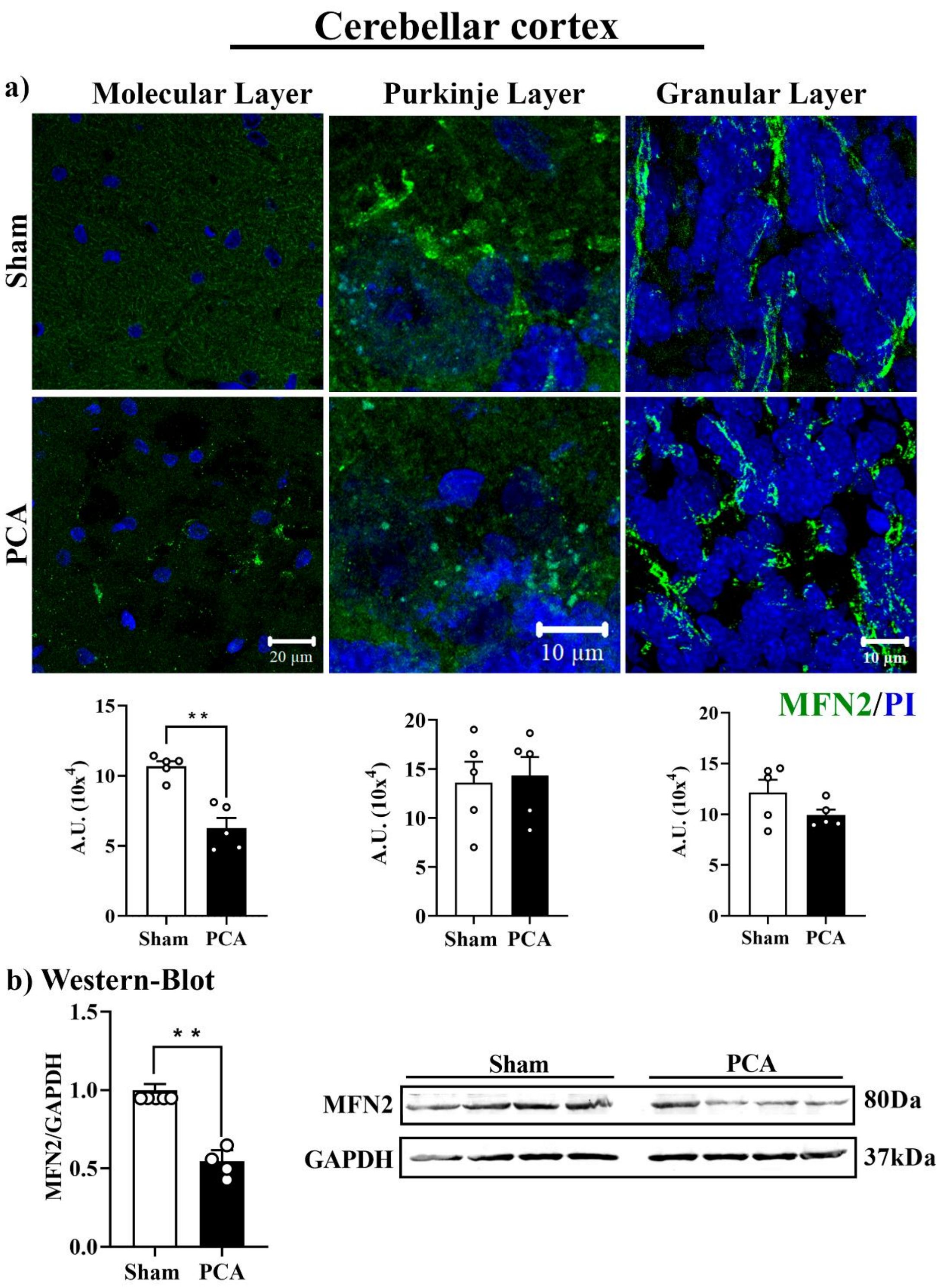

SUPPLEMENTARY FIGURE 4. **Mitofusin 2 (MFN2) protein evaluation in the cerebellar cortex of PCA rats**. a) Representative images in the molecular, Purkinje, and granular layers of Sham rats (top) and PCA rats (bottom). Graphs show the quantification of the fluorescent signal in each cerebellar layer. b) Western-Blot image and densitometric analysis of MFN2

protein expression. Protein levels were normalized to GAPDH expression. Data are reported as mean ± SEM. Significance was calculated with student's *t-test* (**, $p<0.001$), n=5.

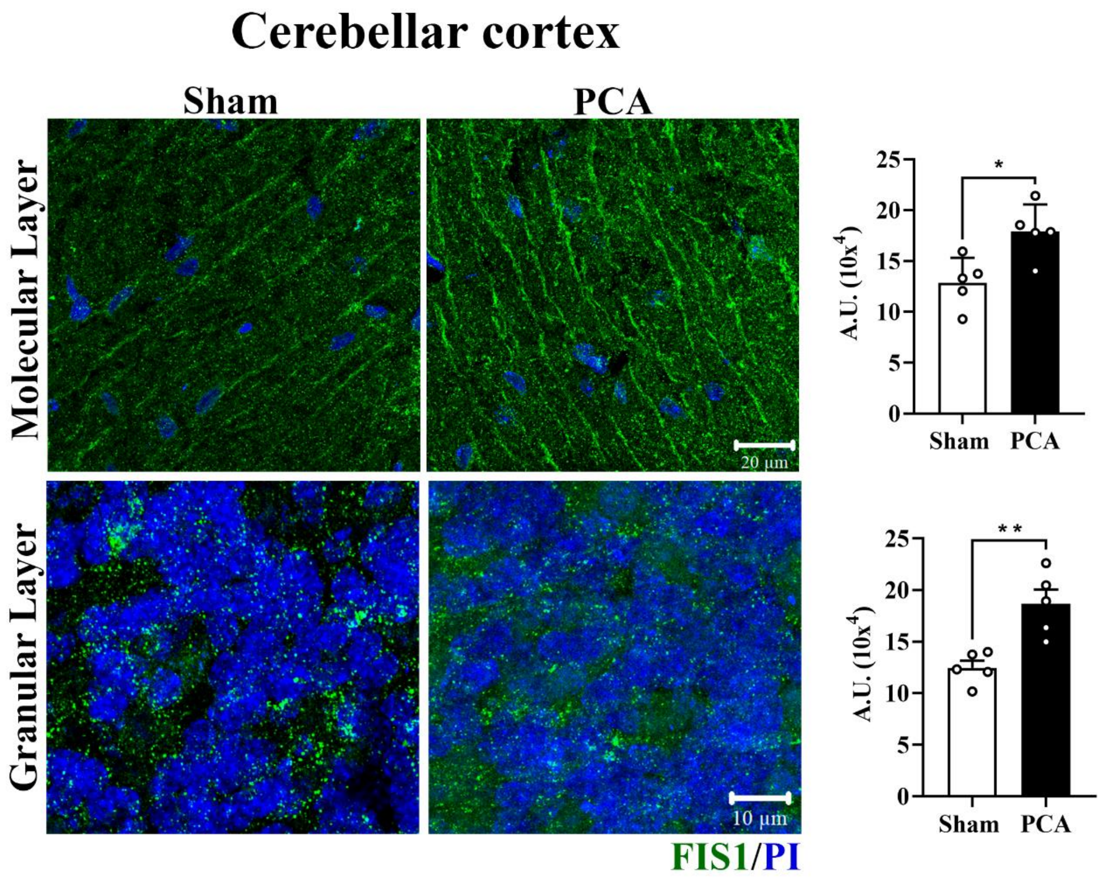

SUPPLEMENTARY FIGURE 5. **Mitochondrial Fission protein 1 (FIS1) evaluation in the molecular and granular layers of PCA rats**. a) Representative images top: in the molecular, bottom: granular layer of Sham rats (left) and PCA rats (right). Graphs show the quantification of the fluorescent signal in each cerebellar layer. b) Western blot image and densitometric analysis of FIS1 protein expression. Protein levels were normalized to GAPDH expression. Data are reported as mean ± SEM. Significance was calculated with student's *t-test* (*, p<0.01; **, p<0.001), n=5.

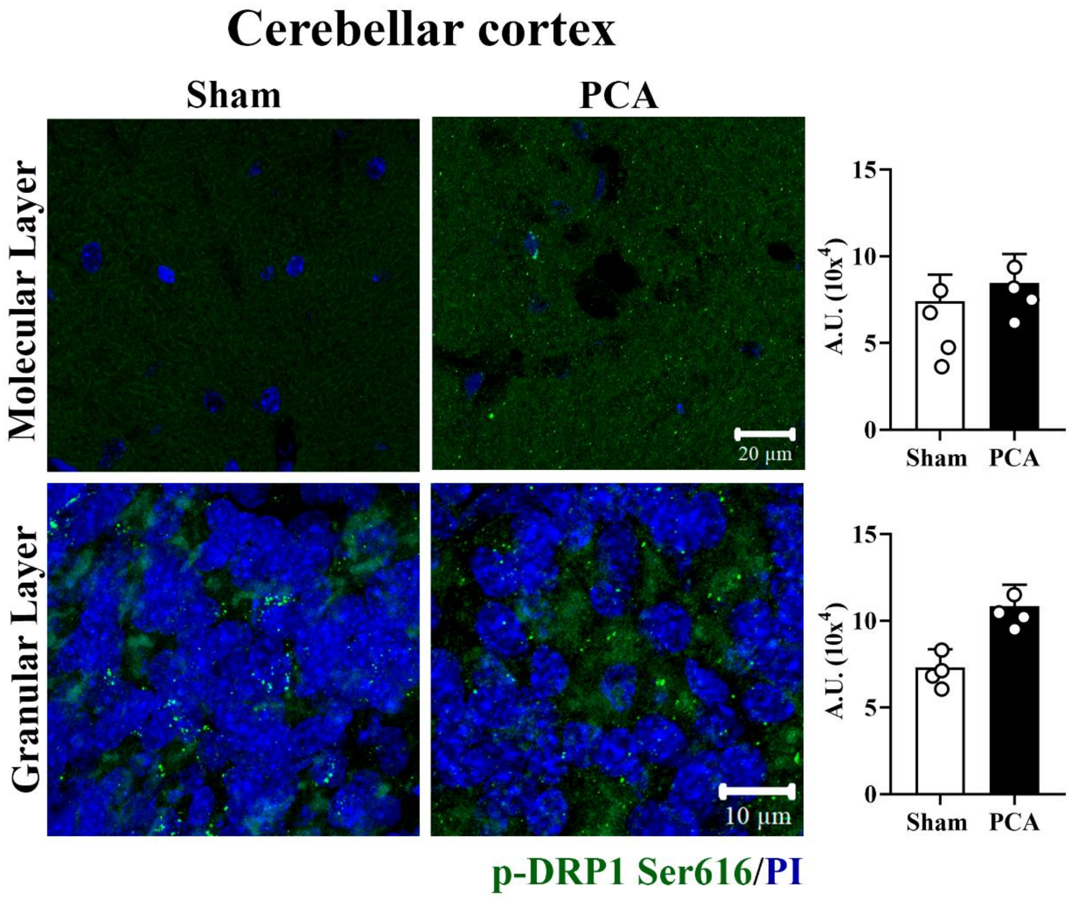

FIGURE 6. SUPPLEMENTARY FIGURE 5. **Dynamin-related protein 1 (DRP1) Phosphorylated (Serine 616) in the molecular and granular layers of PCA rats**. a) Representative images top: in the molecular, bottom: granular layer of Sham rats (left) and PCA rats (right). Graphs show the quantification of the fluorescent signal in each cerebellar layer. b) Western blot image and densitometric analysis of p-DRP1Ser616 protein expression. Protein levels were normalized to GAPDH expression. Data are reported as mean ± SEM. Significance was calculated with student's *t-test*, n=5.
